# Assessing the kinetics of oxygen-unloading from red cells using FlowScore, a flow-cytometric proxy of the functional quality of blood

**DOI:** 10.1016/j.ebiom.2024.105498

**Published:** 2024-12-14

**Authors:** Julija Rabcuka, Peter A. Smethurst, Katharina Dammert, Jarob Saker, Gemma Aran, Geraldine M. Walsh, Joanne C.G. Tan, Margarita Codinach, Ken McTaggart, Denese C. Marks, Stephan J.L. Bakker, Amy McMahon, Emanuele Di Angelantonio, David J. Roberts, Slawomir Blonski, Piotr M. Korczyk, Atsushi Shirakami, Rebecca Cardigan, Pawel Swietach

**Affiliations:** a Department of Physiology, Anatomy & Genetics, University of Oxford, Oxford, UK; b Component Development Laboratory, NHS Blood and Transplant, Cambridge, UK; c Sysmex Europe SE, Bornbarch 1, Norderstedt, 22848, Germany; d Cell Laboratory, Banc de Sang i Teixits, Barcelona, Spain; e Product and Process Development, Canadian Blood Services, Vancouver, Canada; f Research and Development, Australian Red Cross Lifeblood, Sydney, Australia; g Transfusional Medicine Group, Vall d'Hebron Research Institute (VHIR), Universitat Autònoma de Barcelona, Barcelona, Spain; h Product and Process Development, Canadian Blood Services, Ottawa, Canada; i Sydney Medical School, University of Sydney, Camperdown, Australia; j Department of Internal Medicine, University Medical Center Groningen, University of Groningen, Groningen, the Netherlands; k British Heart Foundation Cardiovascular Epidemiology Unit, Department of Public Health & Primary Care, University of Cambridge, UK; l Victor Phillip Dahdaleh Heart and Lung Research Institute, University of Cambridge, Cambridge, UK; m National Institute for Health and Care Research Blood and Transplant Research Unit in Donor Health and Behaviour, University of Cambridge, Cambridge, UK; n British Heart Foundation Centre of Research Excellence, University of Cambridge, Cambridge, UK; o Health Data Research UK Cambridge, Wellcome Genome Campus and University of Cambridge, Cambridge, UK; p Health Data Science Research Centre, Human Technopole, Milan, Italy; q NHS Blood and Transplant, John Radcliffe Hospital, Oxford, UK; r Radcliffe Department of Medicine, University of Oxford, Oxford, UK; s Institute of Fundamental Technological Research Polish Academy of Sciences, Warsaw, Poland; t Medical and Scientific Affairs, Sysmex Corporation, Kobe, Japan; u Department of Haematology, University of Cambridge, UK

**Keywords:** Haematology, Erythrocytes, Storage lesion, Assay, Oxygen transport, Transfusion

## Abstract

**Background:**

Metrics evaluating the functional quality of red blood cells (RBCs) must consider their role in oxygen delivery. Whereas oxygen-carrying capacity is routinely reported using haemoglobin assays, the rate of oxygen exchange is not measured, yet also important for tissue oxygenation. Since oxygen-unloading depends on the diffusion pathlength inside RBCs, cell geometry offers a plausible surrogate.

**Methods:**

We related the time-constant of oxygen-unloading (τ), measured using single-cell oxygen saturation imaging, with flow-cytometric variables recorded on a haematology analyser. Experiments compared freshly-drawn RBCs with stored RBCs, wherein metabolic run-down and spherical remodelling hinder oxygen unloading.

**Findings:**

Multivariable regression related τ to a ratio of side- and forward-scatter, referred to herein as FlowScore. FlowScore was able to distinguish, with sensitivity and specificity >80%, freshly drawn blood from blood that underwent storage-related kinetic attrition in O_2_-handling. Moreover, FlowScore predicted τ restoration upon biochemical rejuvenation of stored blood. Since RBC geometry and metabolic state are related, variants of FlowScore estimated [ATP] and [2,3-diphosphoglycerate]. The veracity of FlowScore was confirmed by four blood-banking systems (Australia, Canada, England, Spain). Applying FlowScore to data from the COMPARE study revealed a positive association with the time-delay from sample collection to measurement, which was verified experimentally. The LifeLines dataset revealed age, sex, and smoking among factors affecting FlowScore.

**Interpretation:**

We establish FlowScore as a widely-accessible and cost-effective surrogate of RBC oxygen-unloading kinetics. As a metric of a cellular process that is sensitive to storage and disease, we propose FlowScore as an RBC quality marker for blood-banking and haematology.

**Funding:**

See Acknowledgements.

**Research in context**

**Evidence before this study**

The ability of red blood cells (RBCs) to oxygenate tissues depends on how much oxygen they can store, and how quickly this gaseous cargo can be released at systemic capillaries. The former is measured routinely from haemoglobin assays, and their results have defined anaemic thresholds and informed medical guidelines. However, the latter is a kinetic parameter that is technically challenging to measure because it requires resource-intensive methods, such as our single-cell oxygen saturation imaging approach. Recently, we showed that oxygen release from blood can become ‘diffusion-limited’ (i.e., constrained by release from cells), underscoring the importance of kinetic measurements in a full appraisal of blood, particularly at a time when contemporary anaemic thresholds are deemed inadequate. However, without a widely-accessible and cost-effective surrogate, appraisal of oxygen-unloading remains aspirational. An attractive feature of such a surrogate would be compatibility with flow cytometry which underpins the design of modern haematology analysers. Our literature search conduced on May 21st, 2023, indicated no flow-cytometric surrogates for assessing oxygen-handling function by RBCs.

**Added value of this study**

Our study introduces and validates FlowScore: an algorithm for estimating the rate of oxygen-unloading from RBCs using geometry-sensitive light scattering routinely recorded on haematology analysers. We adopted FlowScore to test stored blood, on the basis that storage lesion slows oxygen release, yet no flow-cytometric readout exists for estimating this clinically-significant process. FlowScore had >80% sensitivity, specificity, and accuracy in detecting a decrease in O_2_ unloading rate outside its reference range determined for freshly drawn RBCs. Our algorithm faithfully tracked changes in oxygen-unloading rate, for example, after biochemical rejuvenation of blood. The robustness of FlowScore was verified in four blood banks: Australian Red Cross Lifeblood, Canadian Blood Services, NHS Blood and Transplant (England), and Banc de Sang i Teixits (Spain). When applied to large datasets of the LifeLines and COMPARE studies, FlowScore identified haematological and lifestyle correlates that can provide insights into the factors affecting O_2_-handling by blood.

**Implications of all the available evidence**

As a metric of oxygen-unloading rate, FlowScore enables global harmonisation in RBC quality assessment. This addresses an unmet need in blood banking for cost-effective surrogates of the storage lesion. Implementation of FlowScore in haematology can potentially identify previously uncharacterised anaemias, where tissue oxygenation is compromised by a kinetic rather than capacity problem of RBCs. FlowScore is readily available on haematology analysers, and a bespoke equation could be derived for equivalent devices.

## Introduction

To meet the body's oxygen demand of at least 250 mL/min, blood must have adequate O_2_-carrying capacity and support rapid O_2_ exchange during the relatively brief capillary transit.^1^^,^^2^ Binding to haemoglobin (Hb) in red blood cells (RBCs) increases the O_2_ content of blood, but further adaptations are necessary to ensure rapid O_2_ loading and unloading. A widely held view^3^, ^4^, ^5^, ^6^ asserts that O_2_ unloading at systemic capillaries is not rate-limiting, thus O_2_ delivery depends only on the rate of blood flow (‘perfusion limitation’). However, O_2_ release down a partial pressure gradient is not instantaneous because it depends on O_2_ unbinding from Hb and O_2_ diffusion through RBC cytoplasm, a process hindered by macromolecular crowding and reversible binding (‘buffering’) to Hb. To quantify O_2_ unloading, we developed single-cell oxygen saturation imaging.^7^, ^8^, ^9^ Our measurements indicated that O_2_ unloading follows a time constant (τ) of ∼1 s, which implicates a greater diffusion barrier than previously believed.^7^, ^8^ The magnitude of this barrier increases in RBCs stored for transfusion, consistent with their spherical remodelling^1^^,^^2^^,^^10^ and depletion of metabolites such as 2,3-diphosphoglycerate (2,3-DPG)^11^ that regulate HbO_2_ stability.^12^^,^^13^ However, this kinetic attrition is donor-unit dependent,^14^, ^15^, ^16^ reflecting donor profile and manufacturing processes. In perfused human kidneys, we demonstrated that slower τ in stored blood reduces respiratory rate and cortical oxygenation,^17^ arguing for diffusion-limited O_2_ delivery. Kinetic attrition during storage may impact transfusion outcomes,^18^ although this remains contentious.^3^, ^4^, ^5^, ^6^

Since τ relates to the metabolic status and morphology of RBCs, it provides a calibrated and intuitive assessment of their physiological quality. Such a readout is desirable for describing storage lesion because blood-banking workflows must meet compliance standards and quality control, particularly when processes are revised. Research efforts to improve storage, such as additives^19^, ^20^, ^21^, ^22^, ^23^, ^24^, ^25^ or hypoxia,^26^, ^27^, ^28^ place further demands on assessing RBC quality. However, measuring τ is challenging because of the resources required for single-cell oxygen saturation imaging. Identifying suitable surrogates, such as factors affecting the O_2_ diffusion barrier, becomes crucial. Since flow cytometers interrogate cellular geometry, we speculate that the information needed for approximating τ is obtainable from haematology analysers. Herein, we present FlowScore, a surrogate of τ based on side-scatter and forward-scatter recorded on XN-type Sysmex Haematology Analysers. Performance tests in four national blood banks verified the robustness of FlowScore. Applying our algorithm to blood-related data repositories revealed correlates, such as age and lifestyle factors. We present FlowScore as a cost-effective and reliable surrogate of RBC O_2_-handling function, useful for blood-banking and haematology, and meta-analyses.

## Methods

### Blood sampling and production

Blood was collected from registered donors of each participating blood agency and handled according to local procedures and industry guidance. At venipuncture, the first ∼20 mL blood is routinely diverted to a pouch, from which tube samples are taken, including EDTA-anticoagulated whole-blood. Samples were transported or stored at room temperature (RT; 20–24 °C) for up to 3 days to test for time-dependent kinetic attrition outside storage-bag conditions. Whole-blood units were collected into citrate phosphate dextrose (CPD) anticoagulant in Macopharma FQE or LQT sets. The Spanish agency sampled at this stage. Blood was then processed to obtain leucocyte-reduced red cell concentrates (RCC) in Saline-Adenine-Glucose-Mannitol (SAGM) typically within the first day (Day 1; D1) after collection (England, Canada, Spain, Australia) or, occasionally, on D2 in jurisdictions operating low-temperature controls to maintain RBC quality (Canada). After processing, a proportion of RCC was sampled into tubes without anticoagulant to monitor yield and process (RBC and residual WBC counts, haematocrit), or with EDTA tubes (Canada). In the English and Spanish sites, RCC samples were also stored for several days at RT to test time-dependent kinetic attrition outside storage-bag conditions. The remaining RCC units were stored at 2–6 °C (apart from brief sterile sampling each week of one study) until expiry (D35 for England, D42 for Australia, Canada, Spain).

### PhIPA treatment

Expired RCC units were treated 5:1 (v/v) with a rejuvenating solution of phosphate, inosine, pyruvate, and adenine (PhIPA) containing 54.5 mM dibasic sodium phosphate (AlfaAesar CAT#11592), 45.1 mM monobasic sodium phosphate (AlfaAesar CAT#11591), 100 mM sodium pyruvate (Sigma–Aldrich CAT#P2256), 100 mM inosine (AlfaAesar CAT#A14459.22), 5 mM adenine (AlfaAesar CAT#A14906), sterilised using a 0.22 μm syringe filter (Millex CAT#SLGP33RS). For additional rejuvenation experiments, one component was omitted at a time and replaced iso-osmotically with NaCl (Sigma–Aldrich CAT#31434). Control units received sham treatment with SAGM: 150 mM NaCl, 1.25 mM adenine, 45 mM glucose (Sigma–Aldrich CAT#G8270), 30 mM d-mannitol (Sigma–Aldrich CAT#M17311). Incubation was at 37 °C for 60 min with periodic mixing. RBCs were pelleted by centrifugation (600× g) and three wash cycles with 25 mL of SAGM, followed by resuspension in 25–30 mL of SAGM to a 55–65% haematocrit.

### Haemolysate absorbance

Venous blood, collected in EDTA vacutainers (BD CAT#367838), was centrifuged (600× g) and RBCs were diluted 10-fold in HEPES-buffered normal Tyrode: 130 mM NaCl, 4.5 mM KCl (Sigma–Aldrich CAT#31248), 1 mM CaCl_2_ (Honeywell CAT#21114), 1 mM MgCl_2_ (Honeywell CAT#63020), 5 mM glucose, 20 mM HEPES (Sigma–Aldrich CAT#H3375), titrated with NaOH to pH 7.4. Diluted RBCs were split, centrifuged and re-suspended in either normal Tyrode or normal Tyrode with 10 mM nitrite (Sigma–Aldrich CAT#S2252) replacing an equivalent [NaCl] to oxidise Hb to methaemoglobin (metHb). Allowing 15 min for oxidation, RBCs were washed twice with normal Tyrode. Residual buffer was aspirated, and RBCs were haemolyzed by resuspending in water and freeze-thawing. Oxyhaemoglobin (oxyHb) and metHb haemolysates were mixed in various ratios and scanned for absorbance (Biotek Cytation 5).

### Haemolysate fluorescence

Haemolysates were prepared as above but using RBCs preloaded with Calcein-AM (Invitrogen CAT#1430) and CellTracker DeepRed (Invitrogen CAT#C34565) at 4 μM and 16 μM, respectively. Various ratios of oxyHb and metHb (final volume 100 μL) were pre-mixed and aliquoted into a black 384-well plate (Greiner CAT#781986) for imaging using the single-cell oxygen saturation microscopy set-up. The integration time for green fluorescence was 6 ms for Calcein and 90 ms for DeepRed fluorescence.

### Single-cell oxygen saturation imaging

O_2_-unloading kinetics were interrogated using our published method.^9^^,^^29^ 1000-fold diluted RBCs were loaded with 4 μM Calcein-AM and 16 μM DeepRed for 10 min, spun, and resuspended with dye-free solution. Cells were aliquoted into a superfusion chamber with a borosilicate-coverslip coated with poly-l-lysine (Sigma–Aldrich CAT#P4707). A valve-operated, gravity-fed system exchanged solutions between an oxygenated reservoir of normal Tyrode (in air) and an anoxic reservoir of solution bubbled with 100% N_2_ gas and supplemented with sodium dithionite (Sigma–Aldrich CAT#71699). Fluorescence, excited at 640 nm and 488 nm, was split at 500–550 nm and 650–700 nm (OptoSplit, Cairns) onto an ORCA Hamamatsu camera with integration times of 200 ms for green and 30–90 ms for red fluorescence to a ratio ∼1. Ratiometric fluorescence was analysed for time constant of deoxygenation using the mean of three technical repeats.

### Haematology analysis and flow cytometry

RBC variables were obtained using laser flow cytometry on the RET-channel of a Sysmex XN-1000 analyser, using CELLPACK DFL fluid. Measurements included RET-RBC-Y (forward-scatter) and RET-RBC-Z (side-scatter). Flow cytometry on a BD LSRFortessa X-20 measured RBCs resuspended either in Ca-/Mg-free PBS or Sysmex CELLPACK DCL, recording 10,000 events/sample on medium flow.

### Statistics

#### Power calculations

To detect a 50% increase in FlowScore/τ from the mean of freshly drawn blood (mean 1.0, SD 0.1),^9^^,^^17^ a sample size (two-sided α = 0.05; 1-β = 0.8) of at least 3 per group is required (G∗Power 3.1). Thus, measurements were performed from at least three biological replicates (donations) and analysed by one- or two-way ANOVA, or Pearson's correlation.

#### Derivation of FlowScore

All RBC-relevant parameters recorded on Sysmex haematology analysers were considered to seek correlations to time constant τ using Pearson's test. Non-linear regression was used to derive an equation for FlowScore from side- and forward scatter data. The sensitivity, specificity and accuracy of FlowScore as a surrogate of τ was determined from a 2 × 2 table using a threshold of the 95th percentile of reference data (freshly drawn RBCs). Significance was determined by chi-square test.

#### Analysis of datasets

COMPARE^30^ dataset was analysed for the relationship between FlowScore and time delay between sampling and measurement, RBC parameters (Pearson's t-test), blood group, age and sex. The LifeLines^31^ dataset were analysed to seek predictors of FlowScore among biological, behavioural, and haematological parameters. Power calculations for sample size were performed in the original publications. For this purpose, all data that included side- and forward-scatter were included. Study participants self-declared their sex assigned at birth, their race and ethnicity. Testing of categorical data used Mann–Whitney U-test for dichotomous variables or one-way ANOVA for polytomous variables. Continuous data were stratified by FlowScore into top and bottom quartiles and tested for significant difference using independent t-test or Mann–Whitney U-test, depending on the normality criterion. Pearson's R for numerical values, Cohen's D for dichotomous categories, or Eta2 for polytomous categories tested for effect-size to quantify the magnitude of differences or strength of association. Due to heteroscedasticity, a polynomial regression model was performed using variables significant at P < 0.05, excluding those with negligible effect-size or intuitively inter-related parameters. Whilst polynomial regression addresses problems that are non-linear, the obtained fits can be more prone to instability and problematic for extrapolations. Fractional polynomials may offer better outcomes, but these are computationally more expensive and their interpretation is less intuitive.

### Mathematical modelling

O_2_-handling was modelled in terms of O_2_, Hb, and HbO_2_ species reacting and diffusing in a cytoplasmic compartment with one spatial dimension.^9^ The HbO_2_ stability constant (KHbO_2_) was modelled as pH-, CO_2_-, 2,3-DPG-, and temperature-sensitive.^32^ HbO_2_ unbinding followed a rate constant of 20 s^−1^, from which the binding constant could be calculated using K_HbO2_. The diffusion-reaction problem was solved over the spatial dimension x = [0, *h*], where *h* is cell thickness for modelling superfusion experiments or half-thickness when modelling circulating cells. The boundary conditions at x = *h* simulated flux with a high O_2_ permeability of 10^4^ μm/s and reflection at x = 0. Hb/HbO_2_ diffusivities were 10 μm^2^/s and O_2_ diffusivity was best-fit to 110 μm^2^/s using data from freshly drawn reference blood.^9^

### Ethics

Whole-blood units and samples were obtained as part of the standard donation processes of each blood agency, where donors had consented for blood to be used for approved research, service development, and quality monitoring purposes. Participants to COMPARE were consented to the trial (ISRCTN90871183) under a process managed by NHSBT and approved by the UK National Research Ethics Service (15/EE/0335).^30^ Participants in the LifeLines study were consented under a process managed by the LifeLines organisation and approved by the medical ethical committee of the University Medical Center Groningen (UMCG METc 2007/152).^33^ Donors/participants were anonymised to the analytical team.

### Role of funders

Some authors were employed by Sysmex Corporation during the time of the study (see Declaration of interests for details). Sysmex Corporation provided research material to the blood agencies involved in the collaboration. Sysmex Corporation and the funding sources had no role in study design, data collection, data analyses, interpretation, or writing of the report.

## Results

### Validation of the method for measuring O_2_ unloading

**Fig. 1:**
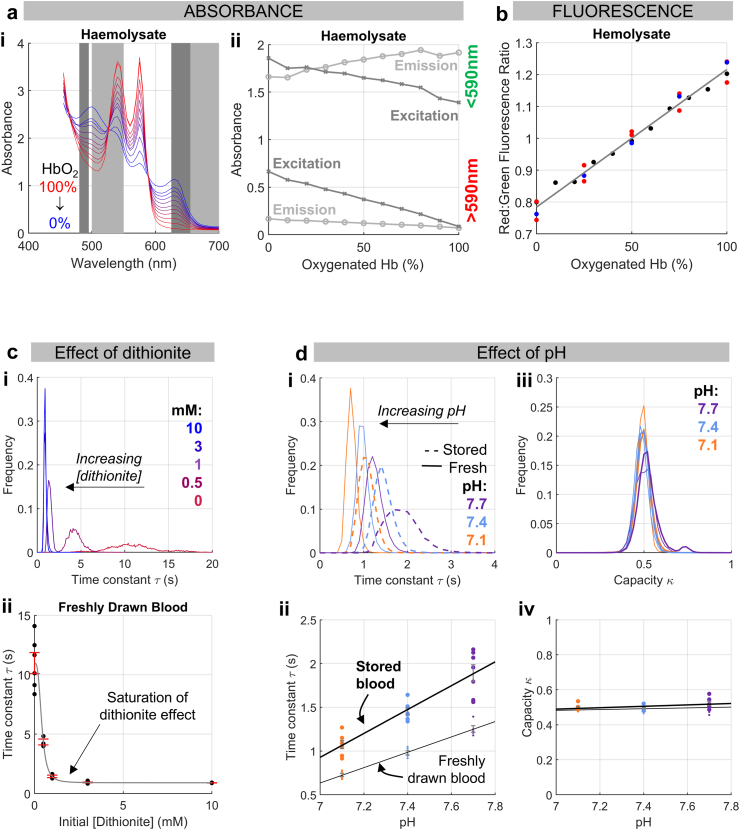
**Validating single-cell oxygen saturation imaging.** (a) (i) Absorbance spectra of haemolysates prepared from a range of ratios of oxyHb and metHb (produced by nitrite treatment). Dark/light grey bands indicate excitation/emission wavelengths used in ratiometric fluorescence imaging. (ii) Haemolysate absorbance as a function of oxyHb concentration, measured at the excitation (dark grey) and emission (light grey) wavelengths for the green (<590 nm) and red (>590 nm) range. Mean (shown for clarity) of 3 biological replicates. Error bars not shown for clarity. (b) The red/green fluorescence ratio is a linear function of the percentage of oxyHb in haemolysates prepared from RBCs that had been loaded with Calcein and CellTracker DeepRed. Imaging followed the same pathlength as for single-cell oxygen saturation imaging. Each colour indicates a biological replicate (three independent blood donations). (c) Effect of increasing [dithionite] in the N_2_-bubbled solution on the time constant (τ) of O_2_ unloading. (i) Frequency distributions, pooled from 6 blood donations (biological replicates). (ii) Concentration-dependence showing saturation of dithionite effect at >2 mM. Mean ± SEM (N = 6). (d) Effect of pH on the time constant of O_2_ unloading. Experiments (N = 5 biological replicates for fresh and 5 biological replicates for stored bloods) on freshly drawn (solid lines) and expired blood unit stored under NHSBT protocols (dashed line) with colour-coding indicating pH. (i) Histograms showing pooled data. (ii) pH-dependence with best-fit line. (iii) and (iv) No change in O_2_-carrying capacity.

Prior to seeking surrogates for the rate of O_2_ release from RBCs, we tested single-cell oxygen saturation imaging against criteria of robustness. The first criterion is linearity between RBC oxygenation and measured fluorescence ratio. Our method uses Calcein- and DeepRed-loaded RBCs to calculate τ by tracking oxygenation from the degree of fluorescence absorbed by Hb; for this to be accurate, the readout must be linearly related to Hb oxygenation (HbO_2_). The O_2_-dependence of absorbance was studied by mixing haemolysates of oxygenated Hb (oxyHb) with metHb, an oxidised derivative prepared by nitrite treatment that has similar spectral properties to deoxyHb. Absorbance at the excitation wavelengths of Calcein and DeepRed decreased with deoxygenation; whereas absorbance at emission wavelengths increased for Calcein but decreased for DeepRed (Fig. 1a). To measure impact on fluorescence, haemolysates were prepared from RBCs pre-loaded with dyes. To generate deoxygenated haemolysate, cells were treated with nitrite prior to lysis, and then mixed with untreated haemolysates. Recordings confirmed that deoxygenation reduces the DeepRed-to-Calcein fluorescence ratio linearly (Fig. 1b). The second criterion is that measured τ is not over-estimated by an artefact of slow mixing between normoxic and anoxic solutions at the point of exchange. Rapid changes to oxygen partial pressure (PO_2_) surrounding cells are enabled by a microfluidic circuit operated by solenoid valves, and further facilitated by using a chemical scavenger, dithionite, to rapidly deplete PO_2_. The relationship between dithionite and τ is expected to plateau once extracellular PO_2_ changes are not rate-limiting for the intracellular deoxygenation process. As shown in Fig. 1c, τ was artefactually slow in the absence of scavenger, but >1 mM, τ in freshly drawn RBCs reached a stable level of ∼1 s, which reports RBC O_2_ unloading kinetics accurately. A third criterion is that τ responds predictably to known regulators of HbO_2_ stability, such as pH. Stronger O_2_ binding to Hb in alkaline cytoplasm is expected to restrict O_2_ diffusion and prolong τ. Moreover, if pathlength is expanded in spherically remodelled cells, a parallel up-shift in the pH-τ relationship is expected. We performed measurements on freshly drawn and stored RBCs, reasoning that the latter have expanded pathlength. Since the relationship between intra- and extracellular pH in RBCs has a slope near unity,^34^ cytoplasmic pH is expected to change proportionately to superfusate pH. The pH-τ relationship was consistent with acidity destabilising HbO_2_ and augmenting O_2_ diffusivity (Fig. 1d). Two-way ANOVA confirmed a significance between pH (P = 10^−12^), of storage (P = 10^−11^), but not of interaction (P > 0.05). The finding that storage causes a parallel-shift to the pH-τ relationship indicates that diffusion pathlength is an important factor determining τ. In contrast, the capacity, κ, of RBCs to carry O_2_ was pH-insensitive.

### Flow-cytometric correlates of O_2_-unloading rate

**Fig. 2:**
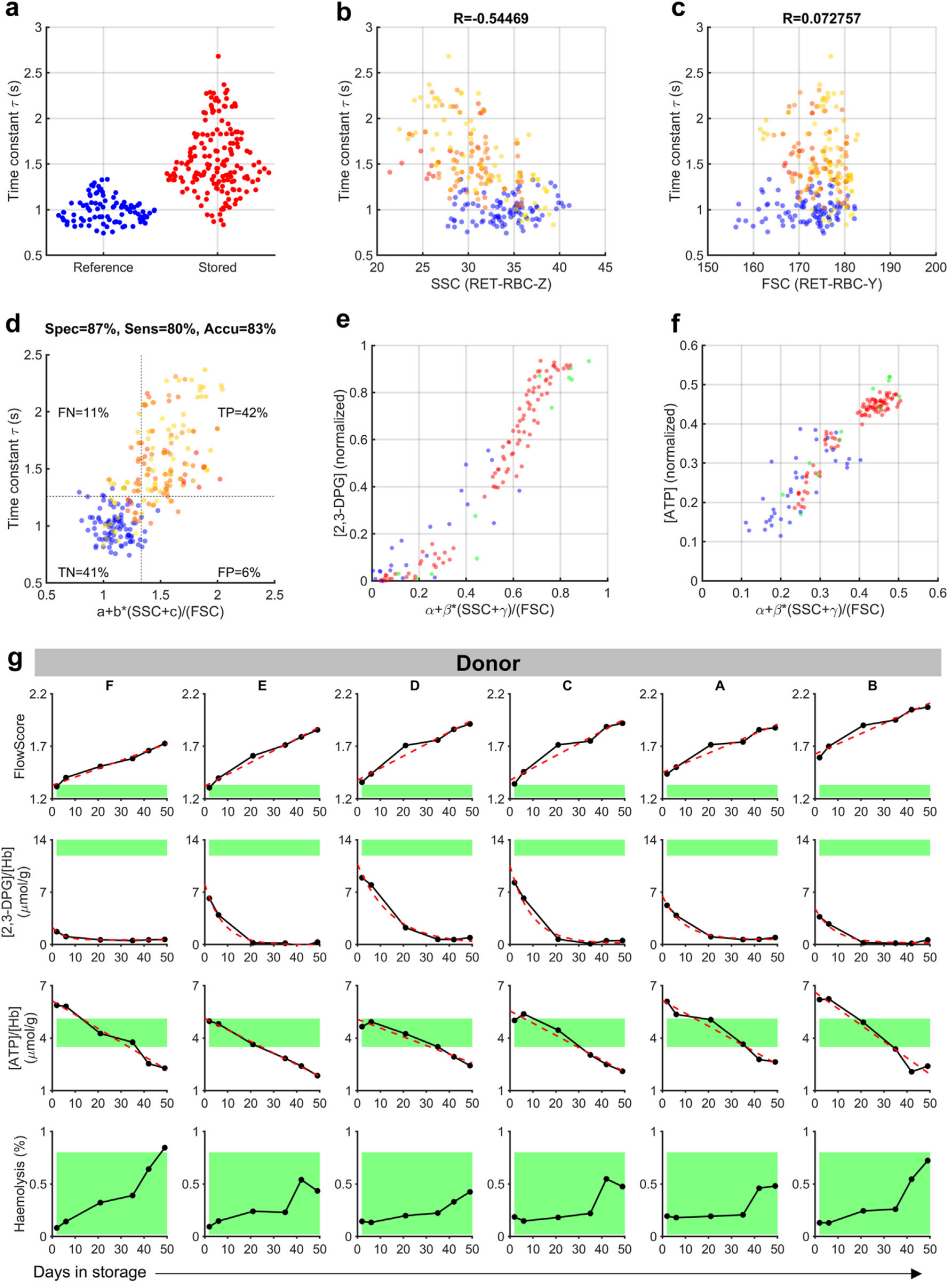
**FlowScore as a surrogate of O**_**2**_**-handling kinetics.** (a) Time constant of O_2_ unloading in freshly drawn blood from registered donors (“reference”; N = 95) and bloods that were stored over a range of durations (2–49 days; N = 165). One-sample Kolmogorov–Smirnov test for normality: P < 10^−10^ for reference and stored blood. (b) Correlations with SSC, showing Pearson's correlation coefficient, R (95% confidence interval: −0.625 to −0.453). (c) Correlations with FSC, showing Pearson's correlation coefficient, R (95% confidence interval: −0.0493 to +0.193). (d) FlowScore coefficients obtained by fitting a non-linear model of SSC and FSC. FN: false negative, FP: false positive, TN: true negative, TP: true positive. Thresholds determined as 95% quantile of reference data. Chi-square test (χ^2^ statistic 115.0012) indicates highly significant correlation (P < 0.00001), with good specificity (spec), sensitivity (sens), and accuracy (accu), greater than 80%. 95% confidence interval for specificity: 81%–93%; sensitivity: 72%–86%. (e) FlowScore variant fitted to [Hb]-normalised [2,3-DPG] or (f) [ATP] obtained from three studies (colour-coded). (g) Temporal evolution of markers of storage lesion in blood units from six donors (A–F). Green range: reference FlowScore, [ATP] or [2,3-DPG] in freshly drawn RBCs, statutory range of haemolysis.

To seek predictors of τ, we analysed Sysmex XN-1000 recordings obtained from our previous studies.^13^, ^17^ Linear regression identified RET-RBC-Z (side-scatter, SSC) as the most significant correlate with τ (Table S1). Since RET-RBC-Y (forward-scatter, FSC) and SSC are measured simultaneously as two aspects of light-scattering from RBCs, both parameters were used to seek a non-linear algorithm that improved correlation with τ. To refine the relationship between τ, SSC and FSC, we combined recordings from RCCs stored under NHSBT-standard conditions (2–49 days)^13^^,^^17^ with new data obtained from RCC units (N = 165) and EDTA whole-blood samples (N = 95) obtained at two NHSBT sites. Each sample was probed for τ (Fig. 2a) and, on the same day, processed for Sysmex XN analysis. τ correlated with SSC (Pearson's test R = −0.54; P = 10^−21^; Fig. 2b) but not with FSC (Fig. 2c). Multivariable regression identified a best-fitting algorithm of SSC and FSC, herein referred to as FlowScore (Fig. 2d) with parameters listed in Table 1:

$$\text{FlowScore}=a+b\times \frac{\text{SSC}+c}{\text{FSC}}\approx \tau$$

**Table 1:** FlowScore parameters for predicting the O_2_ unloading time constant τ, and Hb-normalised [2,3-DPG] and [ATP].

Correlate	Coefficient	Estimate	95% confidence interval		P value
τ	a	7.864	6.553	9.175	<0.0001
	b	−14.41	−16.25	−12.58	<0.0001
	c	46.64	33.25	60.03	<0.0001
[2,3-DPG]/[Hb]	α	−3.577	−4.217	−2.937	<0.0001
	β	15.99	14.91	17.08	<0.0001
	γ	11.42	5.026	17.82	0.00056
[ATP]/[Hb]	α	−1.289	−1.479	−1.10	<0.0001
	β	5.125	4.804	5.447	<0.0001
	γ	23.74	17.708	29.77	<0.0001

The predictive power of FlowScore as an indicator of kinetic attrition in O_2_-handling was tested by setting thresholds at the 95% percentile of FlowScore (1.331 s) and τ (1.259 s) in reference blood. This analysis indicated favourable specificity (87%), sensitivity (80%), and accuracy (83%) to differentiate freshly drawn RBCs from stored cells with slower O_2_ unloading rates (Fig. 2d). The correlation between FlowScore, a descriptor of RBC shape, and τ is explained by diffusion pathlength being an important determinant of O_2_ unloading. Since maintenance of RBC shape is subservient to metabolic state, FlowScore is expected to correlate with metabolite rundown. We investigated this by comparing Hb-normalised [2,3-DPG] and [ATP] with SSC and FSC obtained from two published studies (N = 143).^13^^,^^35^ Multivariable regression described best-fitting coefficients for metabolic variants of FlowScore (Fig. 2e and f; Table 1):

$$\text{FlowScore}=\alpha +\beta \;\times \;\frac{\text{SSC}+\gamma }{\text{FSC}}\approx \frac{\left[\text{metabolite}\right]}{\left[\text{Hb}\right]}$$

The geometrical features interrogated by FlowScore relate to light-scattering measured at two distinct angles. These parameters are available on most flow cytometers, and their ability to detect nuances of RBC shape relate to the non-parallel arrangement of detectors. Sensitivity to RBC shape can be illustrated using osmotic manoeuvres, where solution NaCl is raised or lowered. Since Sysmex analysers are restricted to consumables provided by the manufacturer, the effect of osmolarity was tested on a LSRFortessa research-grade flow cytometer using cells from expired RCC units. As shown in Figure S1, forward scatter increased with hypotonic swelling, consistent with this being a measure of cell size, whereas side scatter decreased, as expected from cells attaining a more spherical shape. In principle, an analogous metric to FlowScore can be calculated for any flow cytometric device with a pair of light scatter detectors. To test this, we paired SSC and FSC recordings obtained on the Sysmex XN-1000 device to those on a LSRFortessa cell analyser, using Sysmex QC standards suspended in either Ca-free/Mg-free PBS or Sysmex CELLPACK DCL (Figure S2). Regression analysis was used to best-fit LSRFortessa-recorded SSC and FSC against paired Sysmex-derived FlowScore. Standards resuspended in PBS and measured in terms of signal height produced the best goodness of fit. These analyses demonstrate that analogous metrics can be derived for other instruments and sample preparations, although these would not be widely comparable without the standardisation achieved for haematology analysers.

### Comparing FlowScore to markers of storage lesion

Markers of storage lesion—notably [2,3-DPG], [ATP], and haemolysis—guide blood-banking practice and improvements to materials and methods. To assess the performance of FlowScore as a marker of storage lesion, the flow-cytometric surrogate was calculated from published^13^^,^^29^^,^^35^ datasets and compared against the three established markers. RCCs from six donors (Fig. 2g)^13^ showed a dramatic depletion of 2,3-DPG over the first 3 weeks of storage, beyond which measurements provided no further insight into the progression of the storage lesion. In early storage, ATP levels were supra-physiological (supported by a buffering effect, e.g., consumption of 2,3-DPG), and then decreased linearly. Thus, for much of the storage duration, [ATP] remained within the range for fresh blood and associated with acceptable post-transfusion recovery (expected count increment).^11^^,^^36^ Haemolysis markedly accelerated after week 5 of storage. Since these three markers interrogate distinct aspects of storage lesion, close matching of their respective time courses is not expected. Significantly, FlowScore met important criteria for a progressive marker of storage lesion that relates mechanistically to O_2_ transport by RBCs. Unlike [2,3-DPG] or haemolysis but akin to [ATP], FlowScore changed steadily during storage, reflecting the progressive attrition of O_2_ unloading kinetics reported previously.^13^^,^^29^ Unlike [ATP], FlowScore in the early phase of storage was near its reference range and worsened over time. As an ensemble metric of cellular geometry, FlowScore is not expected to strictly match the other markers; indeed, a strong correlation would make it redundant. The slope of the FlowScore time-course correlated significantly with the initial rate of 2,3-DPG depletion (Pearson's test rho = −0.875; P = 0.022) but not ATP depletion (Pearson's test rho = −0.586; P = 0.22), possibly because the latter is heavily buffered. Next, the power of FlowScore to detect an improvement to storage protocol (hypoxic/hypocapnic conditions)^29^ and a rejuvenation intervention^35^ was benchmarked against the three established markers. Hypoxic storage helps to maintain key metabolic and gas-handling indices near their physiological range for the first three weeks of storage.^29^ This protective effect is reflected in raised 2,3-DPG and ATP levels, and consistent with this, FlowScore remained within reference range over this timeframe (Figure S3). Critically, the response of FlowScore during this period matched the effect of hypoxic storage on measured O_2_-unloading kinetics.^29^ FlowScore also captured the effect of rejuvenation treatment, which restored FlowScore and [2,3-DPG] to their reference range and overshot [ATP] (Figure S4).^13^

### RBC properties affecting O_2_ unloading

**Fig. 3:**
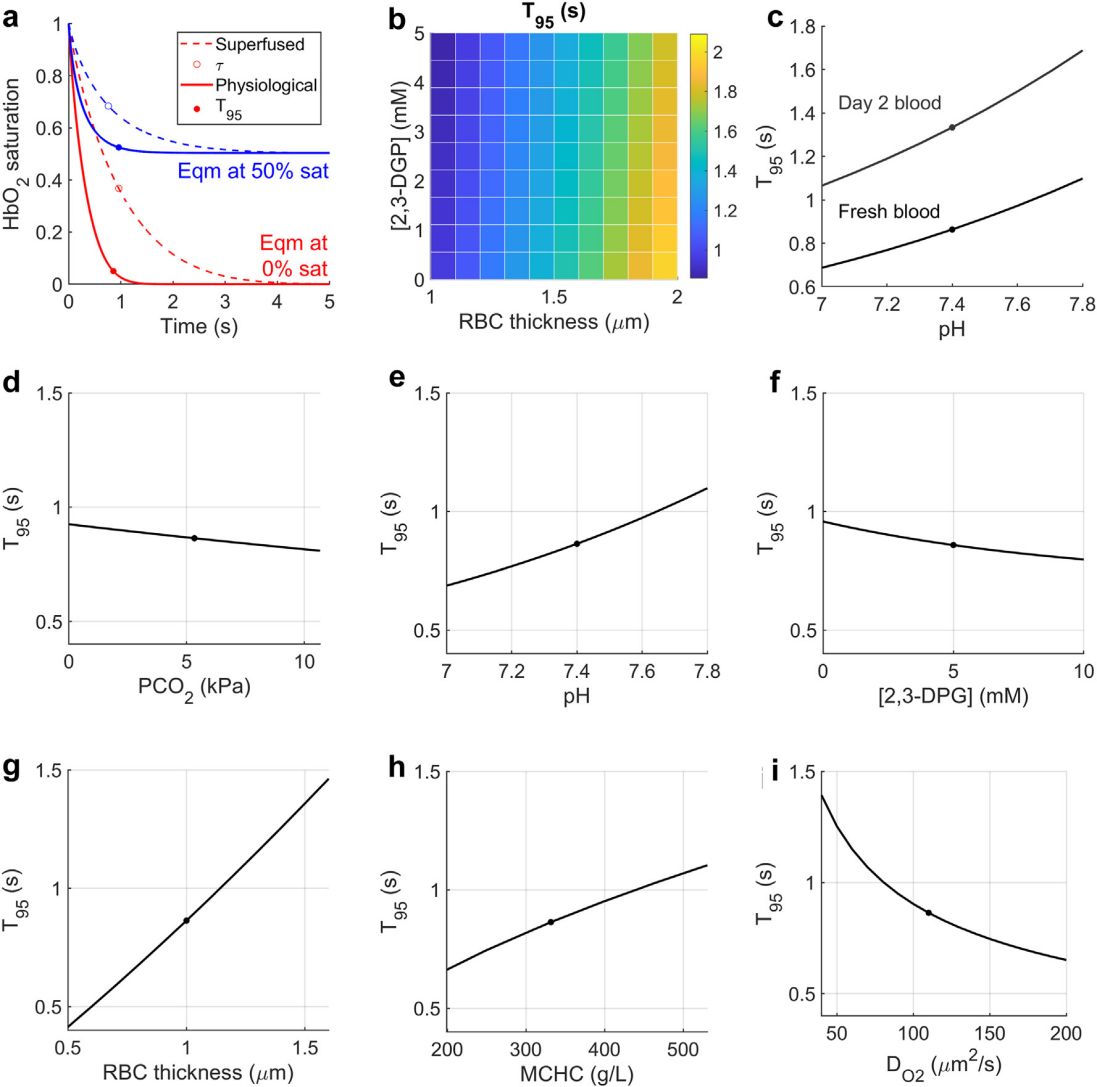
**Factors determining O**_**2**_**-unloading from RBCs.** (a) Mathematical simulation of O_2_ unloading from cells under experimental conditions of single-cell oxygen saturation imaging (dashed line) and physiological conditions (solid line). Open symbol denotes τ (i.e., time constant); filled symbol denotes T_95_ (i.e., time for 95% completion). Two cases were modelled: full O_2_ unloading or 50% O_2_ unloading. (b) Simulating the effect of depleting [2,3-DPG] and increasing cell thickness on τ. (c) Simulating the effect of varying pH in control RBCs and in cells that had been 2,3-DPG depleted and thickened to simulate storage lesion. (d) Modelling the effect on T_95_ of PCO_2_, (e) pH, (f) [2,3-DPG], (g) RBC thickness, (h) mean corpuscular haemoglobin concentration (MCHC), (i) and cytoplasmic O_2_ diffusivity, DO_2_.

O_2_ release from RBCs depends on multiple cellular factors, many of which are difficult to study in isolation. To investigate these dependencies, we performed a sensitivity analysis by mathematical modelling. Since we interrogated O_2_ handling under conditions that optimise control over extracellular PO_2_, track Hb saturation at single-cell resolution, and maximise resolving power,^9^ τ must first be transformed to a measure that relates more closely to circulating cells. For this, we used equations describing the pH-, CO_2_-, [2,3-DPG]-, and temperature dependence of HbO_2_ stability^13^ in a diffusion-reaction model for O_2_ unloading across an intracellular distance of 1 μm, equivalent to RBC half-thickness.^32^ We expressed O_2_ unloading in terms of T_95_, the time-to-release 95% of the RBC's O_2_ content (Fig. 3a). T_95_ increased with a modelled reduction in [2,3-DPG] and with cell thickness, but the latter effect was dominant (Fig. 3b). Simulations of the effect of pH confirmed our experimental findings that low pH reduces T_95_, and that storage causes a near-parallel shift as cells thicken (Fig. 3c). Sensitivity analyses for factors that affect HbO_2_ stability (Fig. 3d–f), for RBC thickness, for mean corpuscular haemoglobin concentration (MCHC) and for intracellular O_2_ diffusivity (DO_2_) (Fig. 3g–i) highlighted diffusion pathlength as a key variable determining O_2_ unloading. These analyses justify the use of FlowScore as a surrogate of O_2_-unloading kinetics.

### Validating FlowScore predictions

**Fig. 4:**
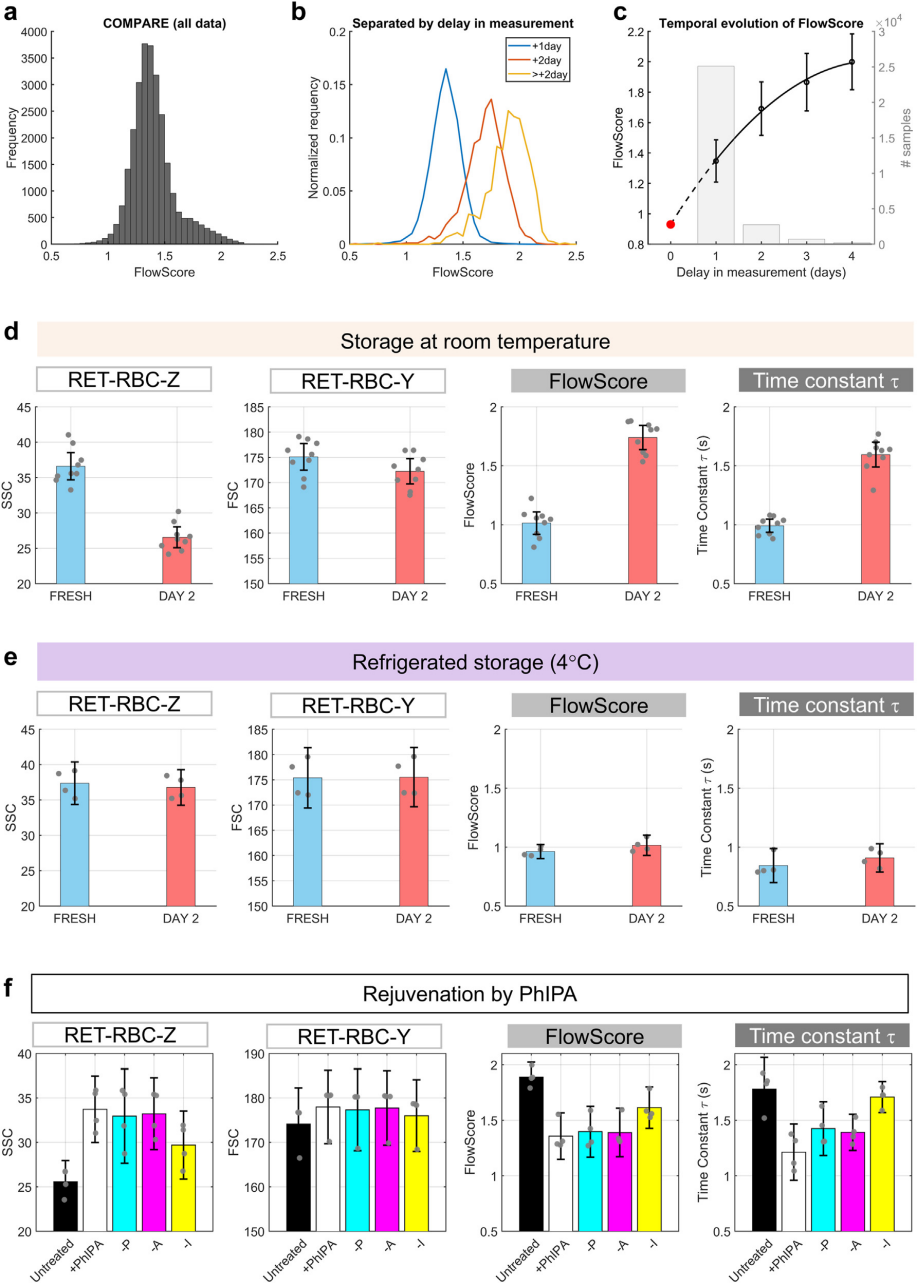
**Validating FlowScore as a surrogate of O**_**2**_**-unloading kinetics.** (a) Distribution of FlowScore calculated from COMPARE study data. (b) FlowScore frequency histograms stratified by time delay between blood sampling and measurement. (c) Temporal evolution of FlowScore (mean ± S.D.) with best-fit extrapolated to measurements on freshly drawn blood (red circle). Bar chart indicates sample size (right axis). (d) Freshly draw blood samples were interrogated immediately for τ (single-cell oxygen saturation imaging), SSC, FSC, and FlowScore (flow-cytometry), or stored for 2 days under room temperature with no additives and then measured for τ, SSC, FSC, and FlowScore. Mean; error bar shows 95% confidence interval (CI) (N = 9 biological replicates; paired experiments). (e) Experiment repeated with storage performed under refrigerated conditions to minimise metabolic rundown. Mean, 95% CI (N = 4 biological replicates; paired experiments). (f) Expired blood units (N = 4 biological replicates) were obtained and split into five groups: receiving no treatment, treatment with all components of PhIPA and treatment with one component omitted at a time. Samples were measured for τ, SSC, FSC, and FlowScore. Mean, 95% CI.

To further test the relationship between τ and FlowScore, we consulted the NIHR BioResource COMPARE study^30^ for FlowScore dependencies that could be verified experimentally for effects on τ. The COMPARE study includes recordings from 29,021 participants, 94% of whom had SSC and FSC measurements. FlowScore was distributed multimodally (Fig. 4a), and its sub-populations relate to different time delays between collection and measurement of samples stored outside blood-banking protocols. Stratifying by delay produced a series of unimodal distributions (Fig. 4b) and extrapolation estimated FlowScore for freshly drawn blood to be ∼1 s, consistent with reference data for τ (Fig. 4c). Of all variables recorded in the COMPARE study, measurement delay had the strongest correlation with FlowScore (Figure S5), indicative of a sensitive marker of storage-related attrition. To verify that measurement delay also affects τ, paired recordings were performed on samples immediately after collection and two days later. To replicate conditions of the COMPARE study, blood samples were kept at room temperature at NHSBT facilities but outside standard storage protocols. Like FlowScore, τ increased after 2 days (Fig. 4d). As a control, the experiment was repeated on refrigerated blood samples to slow metabolic run-down, resulting in unchanged FlowScore and τ (Fig. 4e). The next test for concordance between FlowScore and τ was the effect of biochemical rejuvenation of RCCs using PhIPA.^13^ Paired measurements of FlowScore and τ were made on expired blood units split five ways: receiving no treatment, PhIPA treatment, or one of three variants of PhIPA with one component among pyruvate, inosine or adenine replaced iso-osmotically with NaCl. Phosphate omission was not tested because its absence would compromise pH control. There was a significant recovery of FlowScore and τ following PhIPA treatment, and inosine was deemed the most significant contributor to rejuvenation efficacy (Fig. 4f). The observation that FlowScore and τ responded similarly to rejuvenation confirms the predictive power of our flow-cytometric surrogate.

### Identifying factors affecting FlowScore

**Fig. 5:**
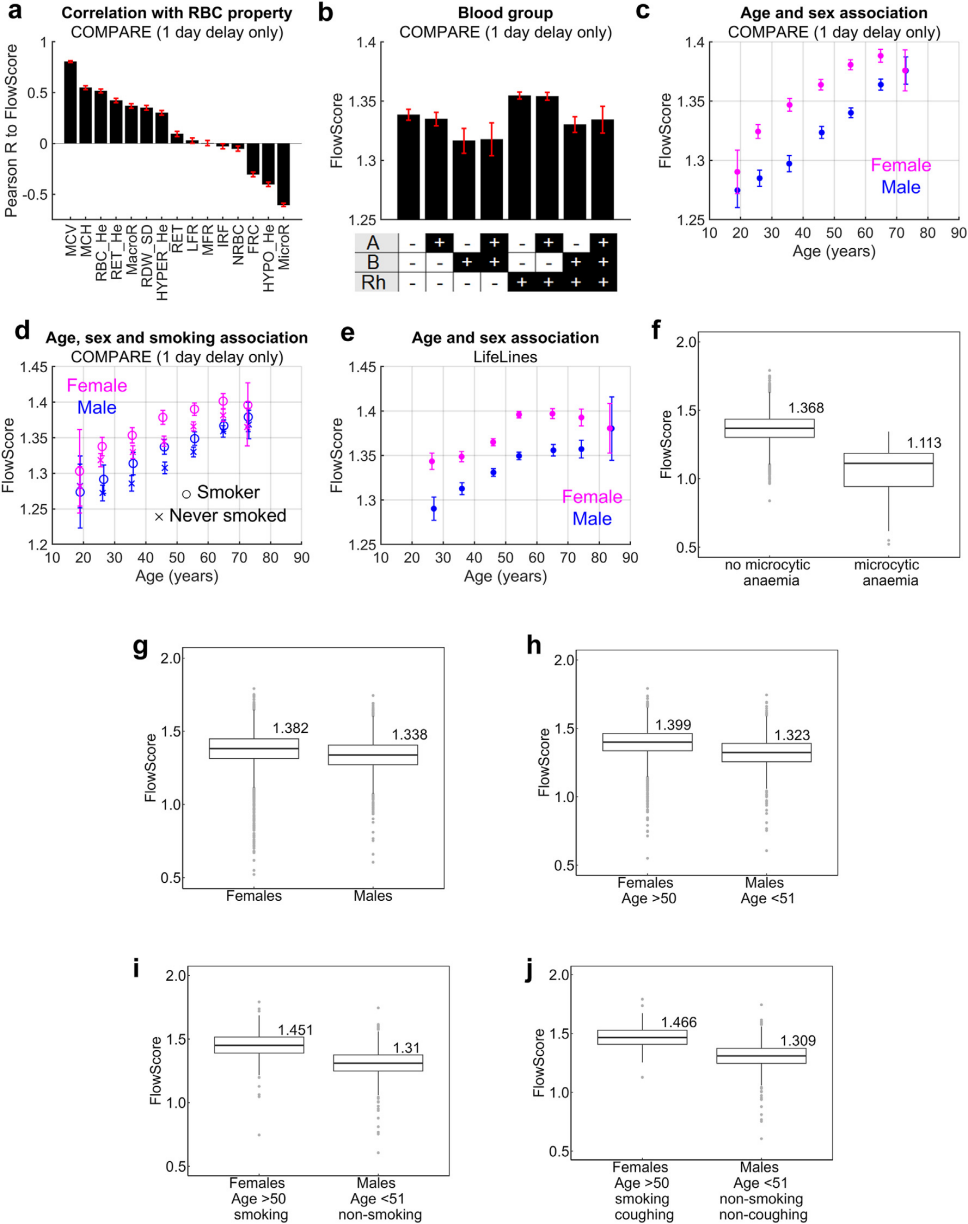
**Analysing COMPARE and LifeLines study data for correlations with FlowScore.** (a) Correlation between FlowScore and RBC parameters annotated with units (Pearson's test). Data obtained after >1 day delay from sampling were excluded. MCV: mean corpuscular volume (fL), MCH: mean corpuscular haemoglobin (pg), RBC_He: haemoglobin content of mature red cells, RET_He: haemoglobin content of reticulocytes, MacroR: percentage of macrocytic RBCs, RDW_SD: red cell distribution width (standard deviation), HYPER-He: percentage of RBC with cellular haemoglobin content higher than 49 pg, RET: percentage of reticulocytes, LFR: percentage of low fluorescence reticulocytes, MFR: percentage of medium fluorescence reticulocytes, IRF: percentage of immature reticulocytes, NRBC: percentage of nucleated RBCs, FRC: percentage of fragmented RBCs, HYPO-He: percentage of RBC with cellular haemoglobin content lower than 17 pg, MicroR: percentage of microcytic RBCs. (b) Relationship between FlowScore and blood group (mean, 95% confidence interval, CI). (c) Relationship between FlowScore and age, stratified by sex assigned at birth (mean, 95% confidence interval, CI). (d) Age-FlowScore relationship stratified by sex and smoking (ever smoked?). (e) Analysis of LifeLines study data. FlowScore and plotted by age and sex (mean, 95% CI). (f) Apparently healthy volunteers (N = 16,028) versus volunteers determined to have microcytic anaemia (N = 190). Outliers represent values outside 1.5× interquartile range from Q1 or Q3. (g)–(j) cumulative effect, on FlowScore, of significant discriminatory factors (sex, age, smoking, coughing). Numbers show median FlowScore. N = 10,090/6833, 5023/3035, 537/2281, 137/2115. Outliers represent values outside 1.5× interquartile range from Q1 or Q3.

Linear regression between FlowScore and RBC-related continuous variables in the COMPARE study identified positive correlations with mean corpuscular volume (MCV) and MCH (Fig. 5a). High macrocytic and low microcytic counts associated with slower FlowScore. Blood group stratified by presence of A-antigen, B-antigen, and Rh factor associated slower FlowScore with the absence of B-antigen and presence of Rh-factor (Fig. 5b). FlowScore increased with age, was slower among females (Fig. 5c), and there was an additive effect of smoking history (Fig. 5d). Further analyses used the LifeLines dataset, a multi-disciplinary prospective population-based cohort study examining the health and health-related behaviours of 167,729 persons in northern Netherlands using a three-generation design^33^^,^^37^ and employs a broad range of investigative procedures in assessing the biomedical, socio-demographic, behavioural, physical, and psychological factors contributing to health and disease. The distribution of FlowScore across the cohort was tailed (Figure S6). Our analyses revealed a significant association with sex, age, smoking, respiratory symptoms, cancer history, snacking and other food-related behaviours, height, alcohol consumption, and joint pain (Table S2 and S3). The relationship between FlowScore and age, stratified by sex (Fig. 5e), was similar to that from the COMPARE dataset. For reference, analysis of the LifeLines dataset indicated faster FlowScore in microcytic anaemia, consistent with its predicted impact of RBC geometry (Fig. 5f). While effect-sizes of individual factors are small, cumulatively they can be meaningful (Fig. 5g–j). The variables affecting FlowScore, determined by a polynomial regression model to account for haematological parameters describing cell shape and haemoglobinization, and after removing confounders, are listed in Table S4.

### FlowScore analytical performance

**Fig. 6:**
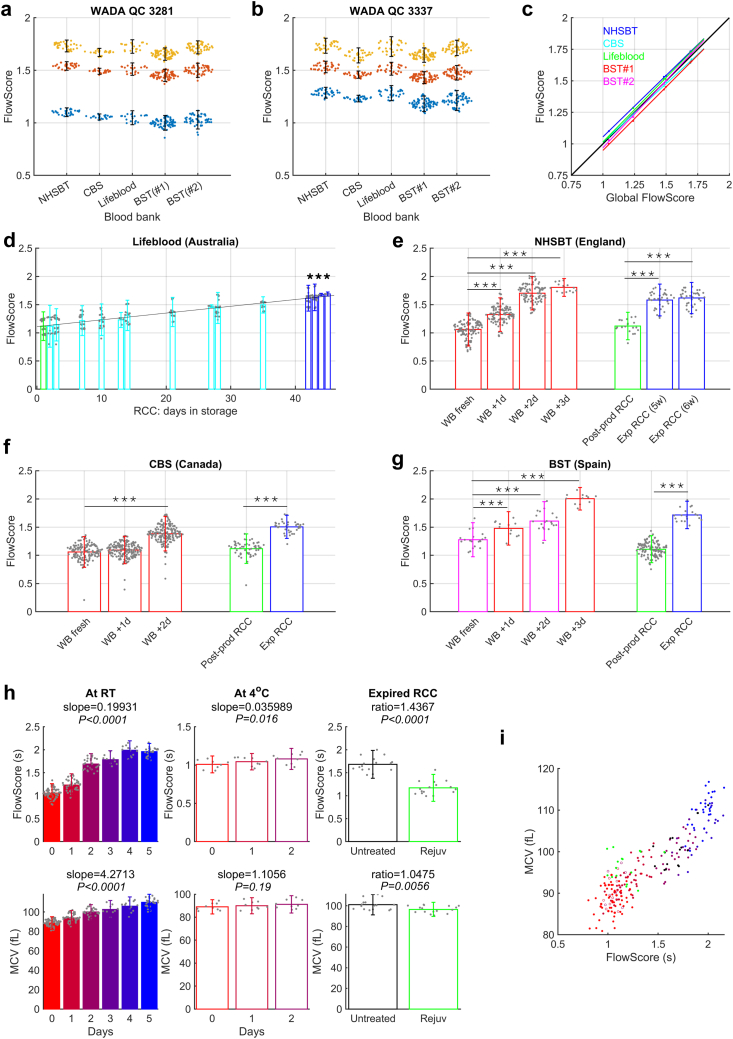
**FlowScore performance analysis.** (a) and (b) Calibration (WADA QC lots #3281 and #3337; each of 3 levels denoted by yellow, red, blue) in four blood banking systems (note; two machines were used in BST). Error bars are 95% confidence interval (CI) around mean. (c) FlowScore relative to “global” mean calculated as average of all blood banks. (d) Data from Lifeblood (Australia). Standard RCC tested over 42-day storage (cyan columns), showing measurements from RCC units post-production (green) and post-expiry (blue). [N = 24, 12, 28, 24, 28, 5, 20, 24, 4, 23, 20, 27, 25, 4, 4] mean, 95% CI. (e) Data from NHSBT (England) [N = 117, 111, 102, 24, 20, 38, 38], mean, 95% CI, (f) CBS (Canada) [N = 200, 193, 199, 104, 56], mean, 95% CI; EDTA whole blood samples measured on the day (usually within 6 h of venipuncture; “WB fresh”) and up to 3 days thereafter (red columns; “WB + 1/2/3d”) and from RCC units post-production (green) or post-expiry (blue). (g) Data from BST (Spain). CPD whole-blood samples (magenta) from unprocessed units on the day of collection (fresh) and after 2 days of RT storage and EDTA whole-blood samples (red) obtained the day after donation (WB + 1d) and after 2 further days of RT storage (WB + 3d). [N = 12, 20, 12, 20, 120, 19], mean, 95% CI. (h) FlowScore (upper) compared to paired measurements of mean corpuscular volume (MCV; lower) for blood sampled at different times at room temperature (RT) (N = 65, 42, 28, 9, 14, 27), under refrigerated conditions (N = 9), or of expired RCC units before and after rejuvenation (N = 16), mean, 95% CI. Confidence intervals for slope/ratio in top row: 0.187–0.212, 0.0072–0.0648, 1.40–1.493; bottom row: 3.95–4.59, −0.574 to 2.785, 1.028–1.067, (i) Relationship between FlowScore and MCV. Colour-coding indicates different experiment, as shown in (h). ∗∗∗ Denotes P < 0.001 (one-way ANOVA; multiple comparisons to fresh blood).

FlowScore must meet performance criteria that include consistent reporting. This was tested using two rounds of calibration with QC standard issued to four blood bank sites: England's NHSBT, Canadian Blood Services, Australian Red Cross Lifeblood, and the Spanish Banc de Sang i Teixits (Fig. 6a and b). FlowScore readings were within 5% error of the global mean (Fig. 6c). FlowScore recordings of RCCs, from production to expiry, are shown for blood at Lifeblood in Fig. 6d. Additionally, each site collected data on RCC post-production and at expiry (Fig. 6d–g). Three sites (England, Spain, Canada) obtained measurements on EDTA whole-blood samples kept for up to 3 days at room temperature to replicate accelerated kinetic attrition (Fig. 6e–g). The Spanish site also obtained data on similarly stored samples of CPD-anticoagulated whole-blood donations prior to processing (Fig. 6g). Post-production RCCs had similar FlowScore to freshly collected blood, and became slower during standard blood-bank storage, reaching comparable levels at expiration in the four national blood banks. The evolution of FlowScore in blood samples stored outside the standard regime followed the trend determined earlier, albeit slower in Canada. Overall, FlowScore reliably conveyed the attrition of RBC physiological quality under storage at four locations. Since cellular geometry is a key factor in determining τ, FlowScore must demonstrably show superior predictive power to other metrics, such as MCV. This was tested in samples subjected to interventions that robustly change O_2_-unloading kinetics: (i) whole-blood storage outside standard protocols at room temperature over 5 days versus refrigeration over 2 days and (ii) biochemical rejuvenation of stored RCCs. Compared to MCV, FlowScore produced stronger and more significant correlations to interventions that affect kinetics (Fig. 6h). Notably, FlowScore was able to resolve the small kinetic attrition under refrigerated storage that evades detection using MCV. Moreover, the effect of rejuvenation was an order of magnitude more prominent on FlowScore, compared to MCV. Notwithstanding the geometric relationships between MCV and FlowScore (Fig. 6i), the latter is a superior metric of kinetic attrition.

## Discussion

### Why is RBC O_2_-handling important to measure?

There is a growing case to record and consider metrics of RBC O_2_-handling when assessing the physiological quality of blood. Two distinct aspects are relevant to this appraisal: (i) the O_2_-carrying capacity of RBCs, inferred from their Hb content and oxygen saturation and (ii) the O_2_-unloading rate from RBCs, which determines the extent of O_2_ release down a PO_2_ gradient during systemic capillary transit. This information is pertinent in the context of transfusion, a common intervention (1.4 million RCC transfused/annum in the UK^38^) for a range of chronic and acute illnesses because restoring oxygen delivery to tissues is the clinical imperative. During storage, RBCs incur a lesion that can cause spherical remodelling, metabolic run-down, and even cell rupture. Whereas the impact of storage on cellular Hb content can be measured from haemoglobinization or haemolysis, the effect of storage on the “kinetic” aspects of O_2_-handling is more difficult to quantify, especially in the setting of blood banks which process large volumes of products and require safe and cost-effective methods. Previously,^13^ we showed that storage can cause RBCs to unload O_2_ more slowly, which is problematic for tissues because their oxygenation may not improve proportionally with blood flow: the physiological strategy for regulating O_2_ delivery. Some evidence for such an effect, related to 2,3-DPG loss, has been noted,^11^ and we recently provided evidence for diffusion-limited exchange in human kidneys *ex vivo* perfused with stored blood before and after rejuvenation.^17^ However, the research methods used to measure cellular O_2_-handling kinetics^9^ are challenging to implement in clinical or blood banking settings.

### How we described FlowScore

To seek cost-effective and accessible markers of O_2_-unloading kinetics (τ), we sought correlations with variables recorded on a widely used haematology analyser, the Sysmex XN. Previously,^13^ we reported that side-scatter is a superior surrogate to τ compared to other markers, such as storage duration, [ATP], or [2,3-DPG]. Using a larger dataset, we refined a non-linear algorithm, called FlowScore, that yields a better estimate of τ by combining side-scatter with forward-scatter. FlowScore was able to distinguish, with sensitivity and specificity better than 80%, storage-related attrition of O_2_-handling. Performance tests in four blood agencies confirmed FlowScore precision and accuracy. Furthermore, FlowScore and τ responded concordantly to biochemical rejuvenation and to an accelerated form of kinetic attrition incurred outside standard storage protocols. To access FlowScore on Sysmex XN analysers, a non-standard module developed for reticulocyte analysis is required. Whilst this is widely available, the appropriate settings must be selected prior to recordings. In principle, similar parameters could be measured on other types of haematology analyser, and an equivalent FlowScore can be described in a product-specific manner. FlowScore sensitivity and specificity may improve by optimising the angle between detectors of side- and forward-scatter. Whilst this is not feasible for haematology analysers on the market, future products may consider such a refinement.

### How is FlowScore related to RBC O_2_-handling?

Our mathematical modelling indicates that O_2_-unloading is exquisitely sensitive to RBC thickness. We postulate that this aspect of geometry is conveyed by FlowScore because the algorithm best-fits two aspects of light scatter to paired τ data. FlowScore had better predictive performance for τ than MCV, a commonly used metric of RBC geometry. Unlike MCV, which is estimated from impedance, FlowScore is sensitive to shape *and* size and therefore better placed to assess the shortest pathlength for O_2_ diffusion. The dumbbell shape of fresh, biconcave RBCs produces a wider side-scatter signal that underpins their fast FlowScore. In contrast, spherically remodelled RBCs produce one smaller side-scatter peak, which is why FlowScore becomes slower. Normalisation to FSC improves signal-to-noise by controlling for an orthogonal measure of scattering and likely explains why FlowScore is stable over time and between sites.

### How is FlowScore related to metabolic status?

Since the maintenance of a biconcave shape ultimately requires an input of energy, RBC geometry is intertwined with metabolic state. This relationship explains why metabolic run-down of ATP and intermediates, such as 2,3-DPG, in stored blood is associated with progressively slower FlowScore. The metabolic run-down that causes cell thickness (diffusion pathlength) to expand synergises with its effect on increasing HbO_2_ stability^10^, ^11^, ^12^, ^13^ that slows O_2_ unloading and restricts its diffusion through an enhanced Hb-buffering effect. A consequence of this metabolic-geometric relationship is that variants of the FlowScore equation correlated with [2,3-DPG] and [ATP]. We therefore demonstrate how FlowScore can be standardised to provide surrogates of a range of inter-related parameters relevant to blood banking. We note that these “metabolite” equations should not be considered as independent or substance-specific surrogates, but as proxies of the ensemble metabolic landscape that impacts O_2_-handling. The ability to approximate RBC metabolic state is timely because it coincides with the discontinuation of a commercial assay kit for 2,3-DPG used in blood banking research and development. A notable advantage of FlowScore over metabolic assays is that it provides information at single-cell resolution, which is critical for determining whether a change in blood quality relates to the removal or introduction of a specific sub-population, or a population-wide change.^39^ For example, FlowScore could provide evidence for changes in microcytic subpopulations that are prone to splenic removal and to depletion with rejuvenation.^39^ A possible limitation of FlowScore may arise when metabolites that normally change in tandem respond differently under specialised storage conditions. This may be relevant to storage under hypoxic conditions, with or without hypocapnia,^29^, ^40^ where the time course of [2,3-DPG] and [ATP] can become altered. It would be important to test whether paired measurements of FlowScore and τ under these conditions fall on the same line of best-fit. Additionally, sensitivity to cell shape may cause FlowScore recordings to be influenced by the tonicity of suspension medium and the analyser diluent.

### What factors affect FlowScore?

As validation of FlowScore robustness, analysis of two independent, large-scale studies (COMPARE and LifeLines) arrived at similar conclusions on the impact of sex and age. The dependency on age may relate to cumulative damage or a shift in the steady-state distribution of RBC geometric properties. Indeed, ageing has been associated with morphological heterogeneity, characterised by increased variation in MCV, with females having a stronger correlation between red cell volume distribution-width (RDW) and age, compared to males.^41^ Slower FlowScore in females is consistent with lower side-scatter reported for LifeLines haematological reference ranges.^42^ Interestingly, MCV reference ranges did not require sex-partitioning,^33^^,^^37^ which indicates that the sex-dependence captured by FlowScore reflects a geometric subtlety. Slower FlowScore in females is expected to negatively impact O_2_ delivery, already reduced by lower haemoglobin levels linked to menstruation. The explanation for the sex-dependence of FlowScore is unclear and beyond the scope of this report, but merits further work. Among all factors available in the datasets, the biggest effect-size on FlowScore was that of microcytic anaemia. In this condition, faster O_2_ unloading from small RBCs may partly offset reduced O_2_-carrying capacity, which we demonstrated for microcytic hypochromic HbH disease.^9^ Fastest FlowScore was associated with AB-/B- blood and slowest in O+/A+ blood, but further work is needed to investigate the underlying mechanisms. The effect of Rh status may relate to the conductivity properties of RhD, a putative NH_3_ and CO_2_ channel,^43^^,^^44^ and a recently described relationship between Rh status and 2,3-DPG levels.^45^ Higher permeability in Rh factor-positive RBCs, or merely the presence of additional integral properties as anchoring sites for the cytoskeleton,^46^ may cause these cells to become thicker and exchange gases more slowly. In line with the notion that O_2_-carrying capacity and O_2_-handling kinetics are distinct parameters, it is notable that blood group does not affect O_2_ saturation.^47^ The discovery of factors affecting O_2_ handling may identify optimal donors and donations that expire more slowly, a step towards personalised transfusion medicine.^48^ Interpretation of FlowScore can impact our understanding of the physiology of athletic performance; for instance, the sex-dependence of FlowScore may explain why arterial hypoxaemia, arising from diffusion-limited pulmonary exchange, is more likely in elite females than elite males.^49^ The use of FlowScore could make a case for “kinetic” anaemia, a disorder that does not meet current guidelines based on Hb thresholds but manifests as reduced O_2_ delivery because of a kinetic defect. It is plausible that certain RBC disorders may compromise O_2_ release, even at unchanged O_2_ content. While our investigation highlights a variety of behavioural and biological factors that impact FlowScore, it must be stressed that due to the design of the dataset, we can only comment on correlation. Some emergent relationships may, for instance, arise because of co-variation and confounding variables. The effects of individual behavioural factors are relatively small and unlikely to be biologically meaningful *per se*, but their superimposition in certain cohorts can result in significant effects on O_2_ handling that will require further research, guided by FlowScore.

### How could blood services use FlowScore?

Quality control of stored blood products is mandated by legislation.^50^^,^^51^ To that end, we envisage FlowScore as a metric of the physiological quality of stored RBCs because O_2_-unloading kinetics are more labile than their O_2_-carrying capacity. Since haematology analysers are widely available, FlowScore is an accessible marker of cellular performance to complement other quality metrics.^11^ Importantly, FlowScore does not require additional sample processing or time-consuming assays. As a continuous variable, we would caution against using a specific cut-off to assign a binary “quality mark” of individual blood donations but encourage the use of its quantitative information. For example, significant shifts in FlowScore between blood banking processes may signal effects on O_2_ unloading and ultimately guide improvements to practice or allocate products to recipients in a needs-basis, e.g., units with fastest FlowScore to be given to those with a clinical need for immediate improvement to oxygen delivery (e.g., in traumatic brain injury) or where transfusion-loads are massive (paediatric intensive care). Taken as a marker of general RBC metabolic state, FlowScore may be useful for assessing post-transfusion recovery.^52^^,^^53^ Between blood agencies, including those in low-income and middle-income countries, FlowScore can rapidly identify pinch-points of product quality and help level-up practice globally. For example, we found that FlowScore is highly sensitive to the delay between sample collection and measurement, indicating significant attrition if blood is not stored under blood bank-grade conditions. This effect may be critical in countries with higher ambient temperatures. Furthermore, any changes that inevitably arise in blood product manufacture, such as the upcoming ban on di-(2-ethylhexyl) phthalate (DEHP) plasticiser in medical devices in Europe, or the introduction of rejuvenation steps, will mandate quality evaluation to ensure that the proposed changes yield a product that is no worse from the original. FlowScore has notable advantages over current markers of storage lesion. Haemolysis, for example, describes the fraction of cells that have succumbed to rupture, but does not provide insight into the quality of the remaining RBCs. FlowScore addresses this limitation by assessing the quality of intact RBCs. Recent efforts, such as those by the Recipient Epidemiology and Donor Evaluation Study (REDS),^54^ are underway to better understand the genetic and non-genetic determinants of storage lesion, which could predict blood product quality and pre-select donors. Whilst there is potential for developing user-friendly look-up algorithms, these rely on adequate donor characterisation, which is likely expensive. Moreover, these outcomes are predictive, whereas FlowScore provides on-demand and responsive metrics that are preferable in the constraints and expectations of blood banks.

In summary, we described a cost-effective and widely accessible surrogate for the O_2_ unloading rate from RBCs, a labile metric of physiological function for which there is currently no equivalent in research, blood-banking, or diagnostic practice. FlowScore provides a basis for relating RBC O_2_-handling with measures of tissue oxygenation and transfusion performance. By disseminating the algorithms for FlowScore, our aim is to initiate a community-driven effort to assess the utility of FlowScore in haematology, transfusion medicine, and blood banking. As more data are generated, the equation for FlowScore is likely to be refined.

## Contributors

PS conceived the project, described FlowScore, performed statistical analyses, and wrote the manuscript. JR performed laboratory studies using single-cell oxygen saturation imaging, Sysmex analysis and LSRFortessa flow cytometry. PAS and RC coordinated research efforts across four participating blood banks. PAS, GA, GMW, JCGT, MC, KMcT, and DCM performed storage studies and arranged Sysmex data collection in the blood agencies. PMK and SB contributed microfluidic chambers for measurements. KD performed statistical analyses of LifeLines data. JS and AS contributed reagents and expertise in haematology analysis. EDA, AMcM, and DJR contributed COMPARE biobank study data. JS provided access to LifeLines study data. PS and RC supervised the project. All authors contributed to revisions of the manuscript. All authors read and approved the final version of the manuscript. JR, PAS, JS, KD, and PS accessed and verified the data.

## Data sharing statement

Original data and instructions on using and implementing FlowScore for Sysmex and similar devices will be made available by the corresponding author upon reasonable request. Investigators using FlowScore algorithms are requested to reference this study. For COMPARE or LifeLines data, see original references for relevant datasets.

## Declaration of interests

KD and JS are employees of Sysmex Europe SE. AS is an employee of the Sysmex Corporation. RC is a SAB member for Terumo BCT and her institution received funding from Sysmex for a project unrelated to this publication. UK Patent pending for FlowScore (submitted by Oxford University Innovation representing PS, JR, PAS, and RC).
