# Supplementary material for: Assessing the kinetics of oxygen-unloading from red cells using FlowScore, a flow-cytometric proxy of the functional quality of blood
Source: eBioMedicine. 2024 Dec 14;111:105498. doi: 10.1016/j.ebiom.2024.105498 (PMC11730303; doi:10.1016/j.ebiom.2024.105498)
Supplement: Tables S1–S4 and Figures S1–S6 [file mmc1.docx]

**SUPPLEMENTARY TABLES**

**Table S1:** Correlation analysis between τ and FSC, SSC, MCV and MCH. Data from Donovan et al, 2022. Pearson’s test.

| **Parameter** | **Rho** | **95% confidence interval of Rho** | | **P value** |
| --- | --- | --- | --- | --- |
| FSC | -0.32514 | -0.75608 | -0.13646 | 0.44 |
| SSC | -0.57250 | -0.86194 | -0.42285 | 0.16 |
| MCH | -0.02353 | -0.58706 | 0.17472 | 0.66 |
| MCV | 0.03564 | 0.00000 | 0.38885 | 0.0032 |

**Table S2**: Impact of genetic and lifestyle categorical factors on FlowScore as discovered by analysis of the Lifelines database. Median difference of polytomous variables show difference of the outermost variables.

| **Variable** | **n** | **Median (IQR)** | **Median difference** | **P value** |
| --- | --- | --- | --- | --- |
| Sex assigned at birth: | | | | |
| Female | 10,711 | 1.382 (0.134) | 0.044 | <0.0001 |
| Male | 7,407 | 1.338 (0.133) |  |  |
| Current smoker: | | | | |
| No | 14,318 | 1.356 (0.133) | -0.046 | <0.0001 |
| Yes | 3,493 | 1.402 (0.136) |  |  |
| Do you have cancer or have had cancer? | | | | |
| Yes | 767 | 1.383 (0.134) | 0.020 | <0.0001 |
| No | 17,052 | 1.363 (0.136) |  |  |
| Do you have chronic obstructive pulmonary disease or emphysema? | | | | |
| Yes | 694 | 1.390 (0.151) | 0.026 | <0.0001 |
| No | 15,487 | 1.364 (0.136) |  |  |
| Do you cough almost every day for a period of three months a year? | | | | |
| Yes | 1,582 | 1.387 (0.140) | 0.024 | <0.0001 |
| No | 14,619 | 1.363 (0.135) |  |  |
| Did you suffer from chest wheezing in the past twelve months? | | | | |
| Yes | 1,789 | 1.385 (0.134) | 0.023 | <0.0001 |
| No | 14,394 | 1.363 (0.135) |  |  |
| Do you cough up mucus every day for period of three months a year? | | | | |
| Yes | 1,307 | 1.385 (0.144) | 0.022 | <0.0001 |
| No | 14,889 | 1.363 (0.135) |  |  |
| How often did you eat small cookies or biscuits in the past month? | | | | |
| Not this month | 1,261 | 1.378 (0.138) | 0.026 | <0.0001 |
| 1 day per month | 428 | 1.378 (0.139) |  |  |
| 2-3 days per month | 1,327 | 1.367 (0.129) |  |  |
| 1 day per week | 1,658 | 1.364 (0.132) |  |  |
| 2-3 days per week | 3,097 | 1.362 (0.135) |  |  |
| 4-5 days per week | 2,433 | 1.361 (0.132) |  |  |
| 6-7 days per week | 2,466 | 1.353 (0.135) |  |  |
| How often did you have painful joints in the past 6 months? | | | | |
| Not at all | 8,041 | 1.360 (0.136) | -0.022 | <0.0001 |
| A few times a month | 4,282 | 1.362 (0.138) |  |  |
| A few times a week | 1,650 | 1.375 (0.127) |  |  |
| Every day | 2,117 | 1.382 (0.138) |  |  |
| Worsening of complaints after physical activity in the past 6 months? | | | | |
| Not at all | 10,107 | 1.360 (0.136) | -0.029 | <0.0001 |
| A few times a month | 4,010 | 1.370 (0.133 |  |  |
| A few times a week | 1,254 | 1.374 (0.136) |  |  |
| Every day | 736 | 1.390 (0.141) |  |  |
| Usage of medicine containing paracetamol in the past year? | | | | |
| None | 2,210 | 1.364 (0.136) | -0.029 | <0.0001 |
| Less than 10 a year | 5,608 | 1.359 (0.134) |  |  |
| Between 10-50 a year | 6,739 | 1.366 (0.137) |  |  |
| Between 50-200 a year | 1,758 | 1.375 (0.134) |  |  |
| More than 200 a year | 377 | 1.392 (0.154) |  |  |

**Table S3**: Impact of genetic and lifestyle numerical factors on FlowScore as discovered by analysis of the Lifelines database.

| **Variable** | **Bottom FlowScore quartile (Q1)** | | **Top FlowScore quartile (Q4)** | | **Median difference** | **P value** |
| --- | --- | --- | --- | --- | --- | --- |
|  | **n** | **Median (IQR)** | **n** | **Median (IQR)** |  |  |
| Age (years) | 4,425 | 44 (14) | 4,462 | 48 (13) | -4.0 | <0.0001 |
| Body length in cm | 4,553 | 177 (14.0) | 4,555 | 171 (11.8) | +6.0 | <0.0001 |
| Consumption of bread products | 3,051 | 112 (60) | 3,203 | 84 (56) | +28.0 | <0.0001 |
| Consumption of alcohol | 3,333 | 17 (30) | 3,302 | 24 (40) | -7.0 | <0.0001 |

**Table S4**: Parameters from the Lifelines database, chosen for the polynomial regression model to predict FlowScore based on lifestyle parameters and hematological results.

| **Variable** | **Coefficient Estimate** | **95% CI** | **P value** |
| --- | --- | --- | --- |
| Intercept | 1.212 | 0.02038 | <0.0001 |
| MCV^1 | 10.542 | 0.51744 | <0.0001 |
| MCV^2 | 0.765 | 0.25284 | <0.0001 |
| RBC-He^1 | -4.734 | 0.38808 | <0.0001 |
| RBC-He^2 | -1.793 | 0.32340 | <0.0001 |
| RBC-He^3 | 1.237 | 0.21364 | <0.0001 |
| MicroR^1 | -0.590 | 0.53900 | 0.032 |
| MicroR^2 | 0.556 | 0.19110 | <0.0001 |
| RDW-SD^1 | 0.823 | 0.23540 | <0.0001 |
| RDW-SD^2 | -0.377 | 0.11486 | <0.0001 |
| RBC | 0.0332 | 0.00416 | <0.0001 |
| Sex: male | -0.274 | 0.00337 | <0.0001 |
| Current smoker: yes | 0.0166 | 0.00319 | <0.0001 |
| Alcohol consumption | 0.00021 | 0.00004 | <0.0001 |
| Age (years) | 0.00021 | 0.00010 | <0.0001 |
| Consumption of bread products | -0.00004 | 0.00002 | 0.00060 |
| Chest wheezing: no | -0.00581 | 0.00382 | 0.0029 |
| Paracetamol: less than 10 a year | -0.00405 | 0.00272 | 0.0036 |
| MCH^2 | -1.060 | 0.31948 | <0.0001 |
| MCH^3 | -0.483 | 0.21952 | <0.0001 |
| Snacks: 2 to 3 days per week | -0.00606 | 0.00453 | 0.0086 |
| Snacks: 4 to5 days per week | -0.00498 | 0.00470 | 0.038 |
| Snacks: 6 to 7 days per week | -0.00898 | 0.00474 | 0.00020 |

**SUPPLEMENTARY FIGURES**

**
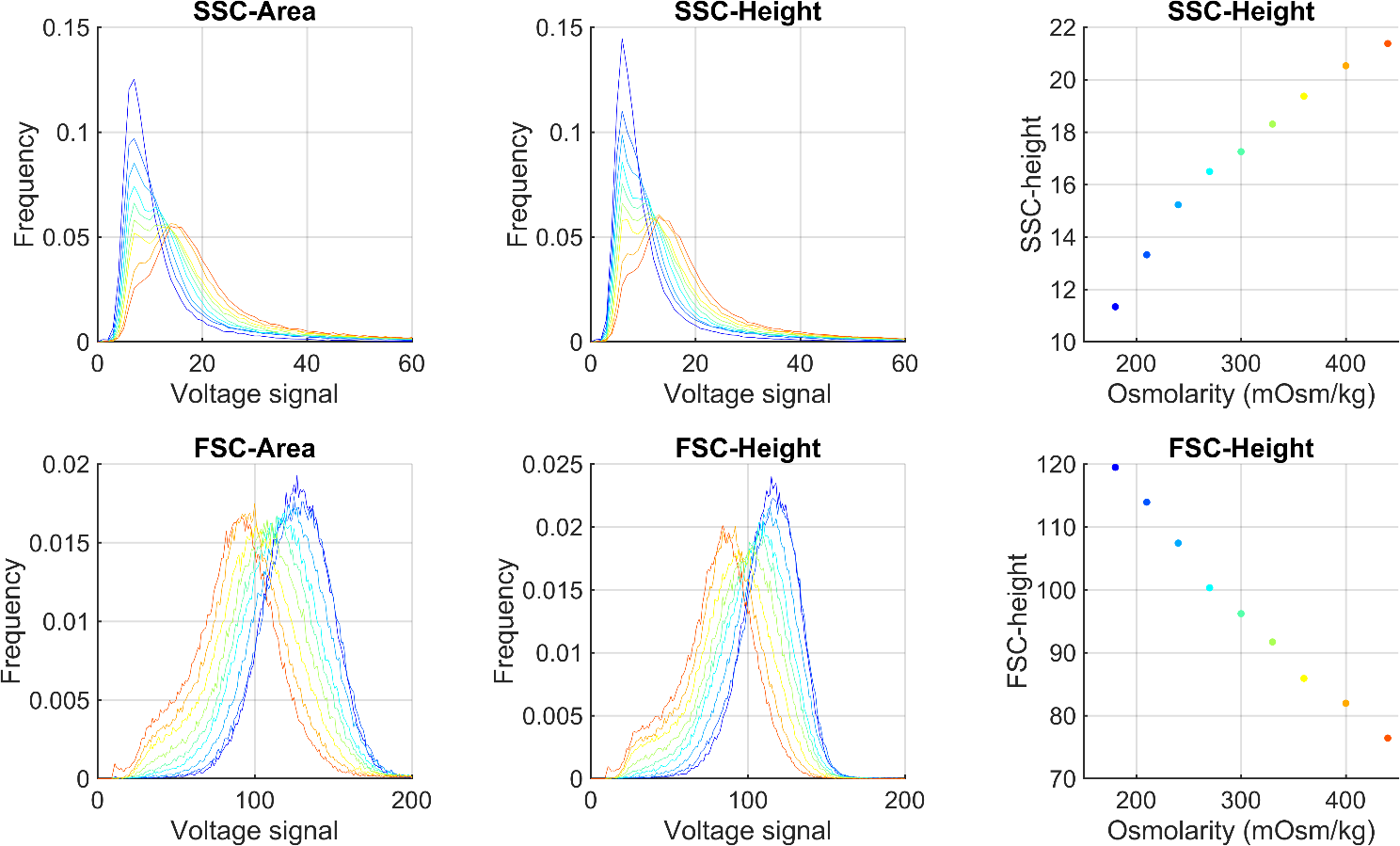
**

**Supplementary Figure S1: Orthogonal responses of side and forward scatter to osmotic cell swelling and shrinkage.** RBCs from expired units were re-suspended in solutions of varying osmolarity (180 to 440 mOsm/kg), attained by altering [NaCl] in normal Tyrode. Measurements of side scatter (SSC; top) and forward scatter (FSC; bottom) were taken using either area or height of signal. Color coding is related to osmolarity. Scatter was recorded as the height or area of the voltage signal.

**
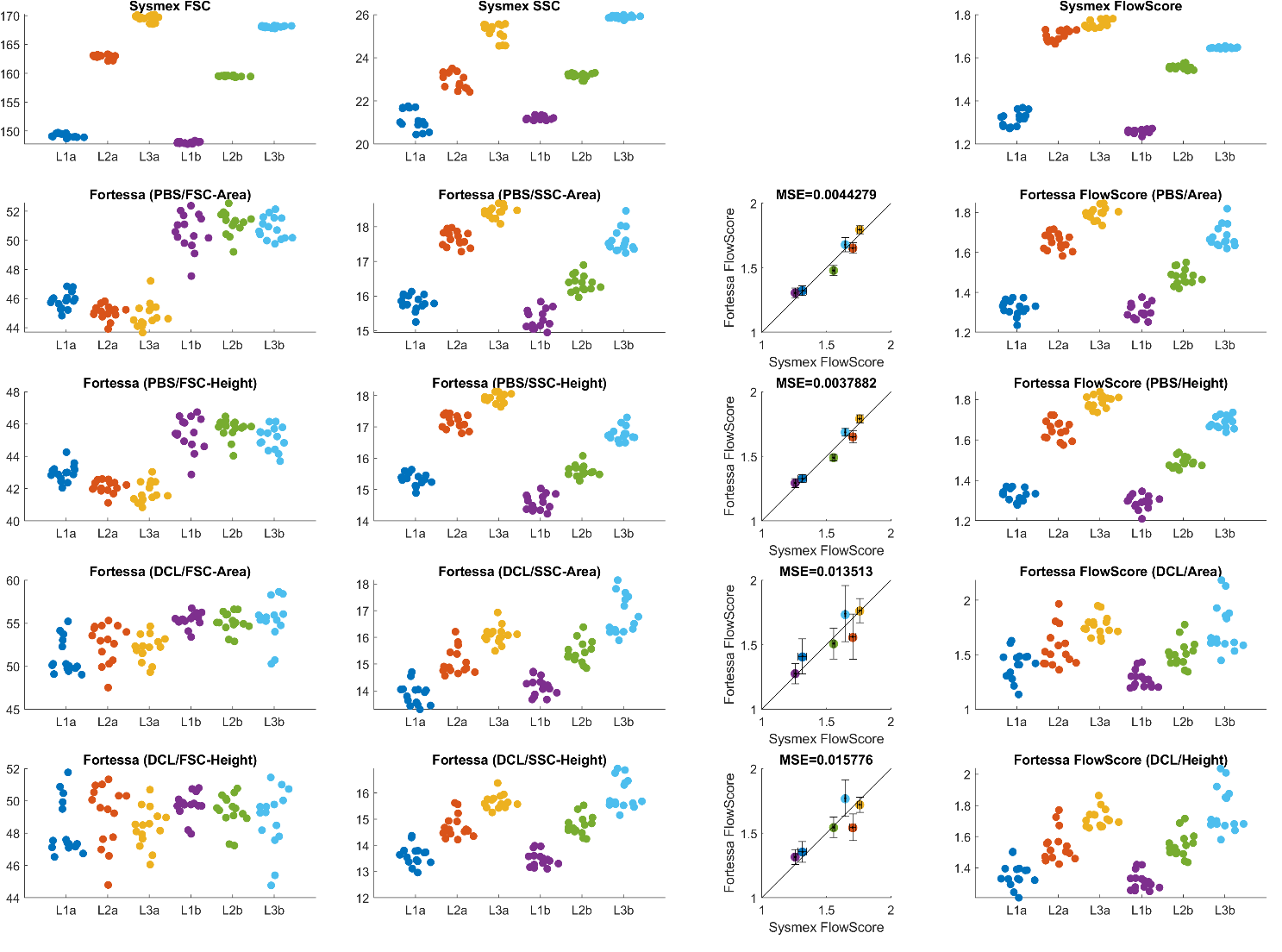
**

**Supplementary Figure S2: Obtaining FlowScore algorithms for research-grade flow cytometers.** Sysmex QC standards (L1-L3) from three batches (a,b) were recorded on a Sysmex XN1000 analyzer and a LSRFortessa research-grade flow cytometer using forward- and side-scatter channels (FSC, SSC) shown in the first and second columns, respectively. The Fortessa analysis used signal height and area independently. QC standards were resuspended in PBS prepared freshly or DCL supplied by Sysmex. Linear regression best-fitted an algorithm of Fortessa-recorded FSC and SSC to the Sysmex FlowScore (third column). MSE indicates mean square error and relates to goodness of fit. Thus, four FlowScore equations could be generated for the Fortessa, of which measurements of scatter height in PBS had best fit and resolving power. Nonetheless, the noise on a Fortessa system is greater than with the Sysmex system. Error bars denote SEM.

Standard storage Hypoxic storage

**
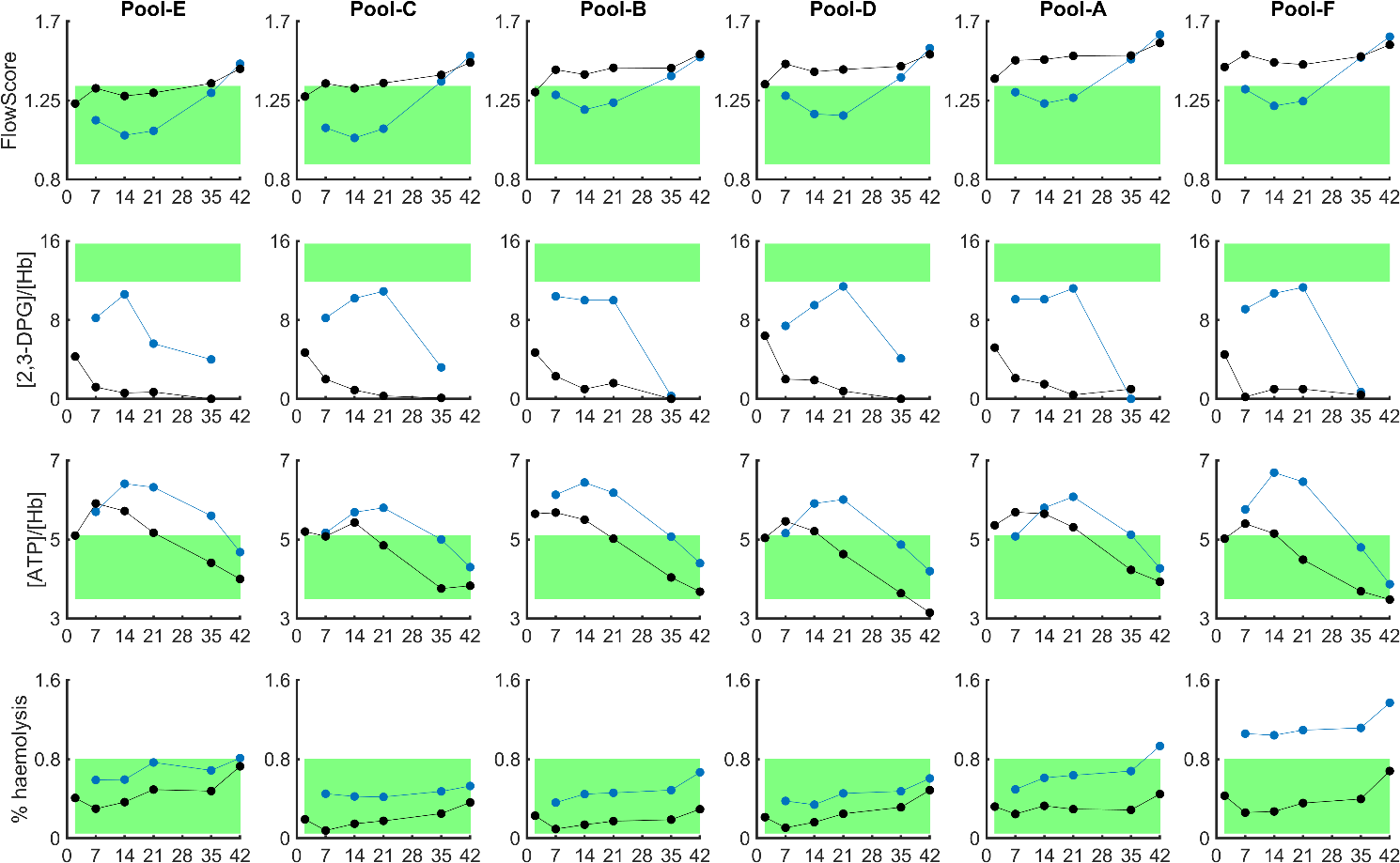
**

**Supplementary Figure S3: FlowScore relative to other markers of storage lesion under standard and hypoxic/hypocapnic storage.** Temporal evolution of markers of storage lesion in six pooled blood units: FlowScore, [2,3-DPG]/[Hb] (μmol/g), [ATP]/[Hb] (μmol/g), % haemolysis. Green range: reference FlowScore (from Figure 2d), [ATP] or [2,3-DPG] in freshly drawn RBCs (35), statutory range of haemolysis (50,51). Pools split for standard (black datapoints) or hypoxic/hypercapnic storage (blue datapoints).

Standard storage Rejuvenated

**
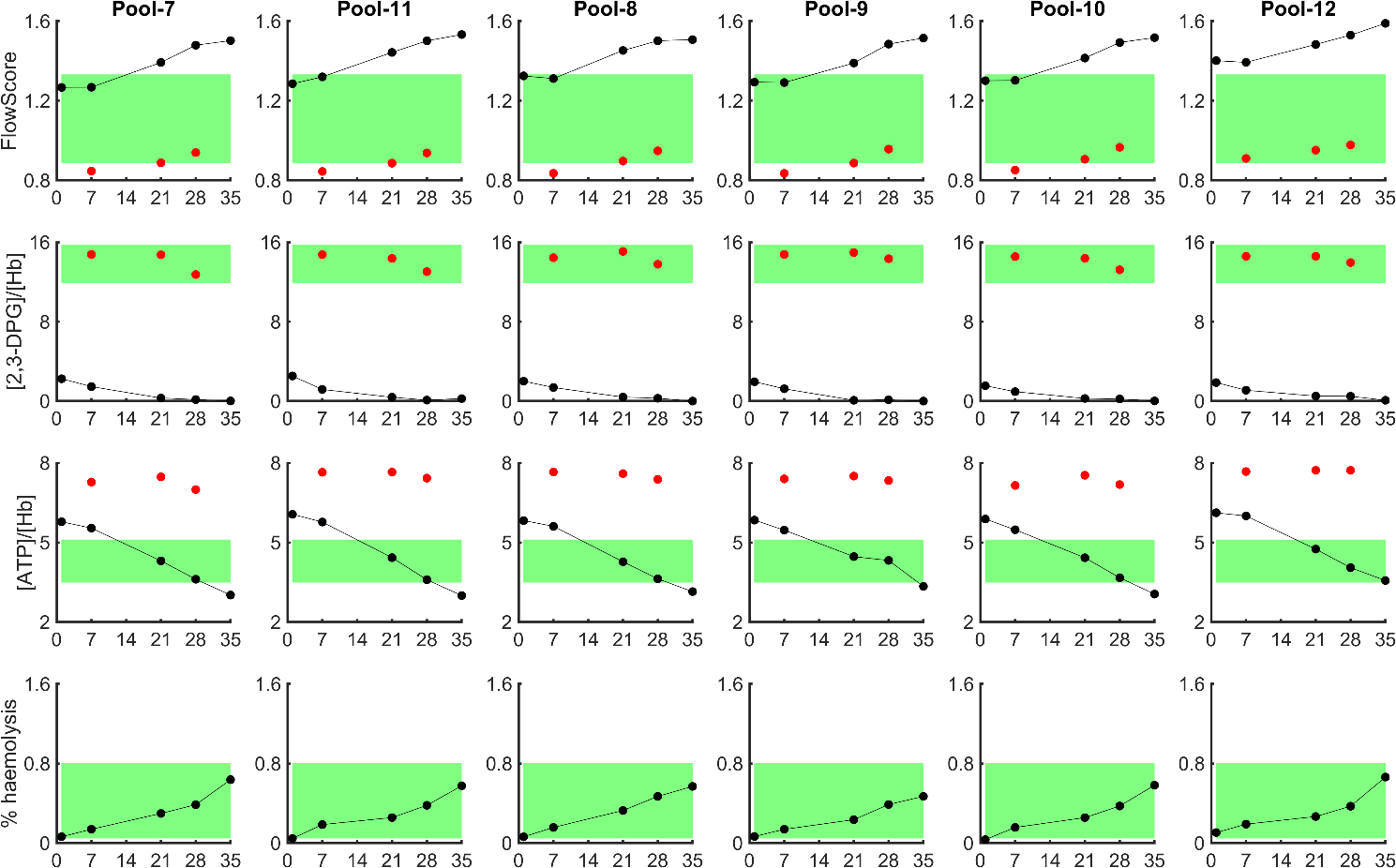
**

**Supplementary Figure S4: FlowScore relative to other markers of storage lesion under standard storage and after rejuvenation.** Temporal evolution of markers of storage lesion in six pooled blood units: FlowScore, [2,3-DPG]/[Hb] (μmol/g), [ATP]/[Hb] (μmol/g), % haemolysis. Green range: reference FlowScore (from Figure 2d), [ATP] or [2,3-DPG] in freshly drawn RBCs (35), statutory range of haemolysis (50,51). Pools under standard storage (black) were split and rejuvenated by PhIPA treatment at the days indicated (red datapoints). Since rejuvenation includes a washing step that replaces the storage supernatant with fresh additive solution, a post-rejuvenation haemolysis value would not reflect storage quality and is therefore not shown.

**
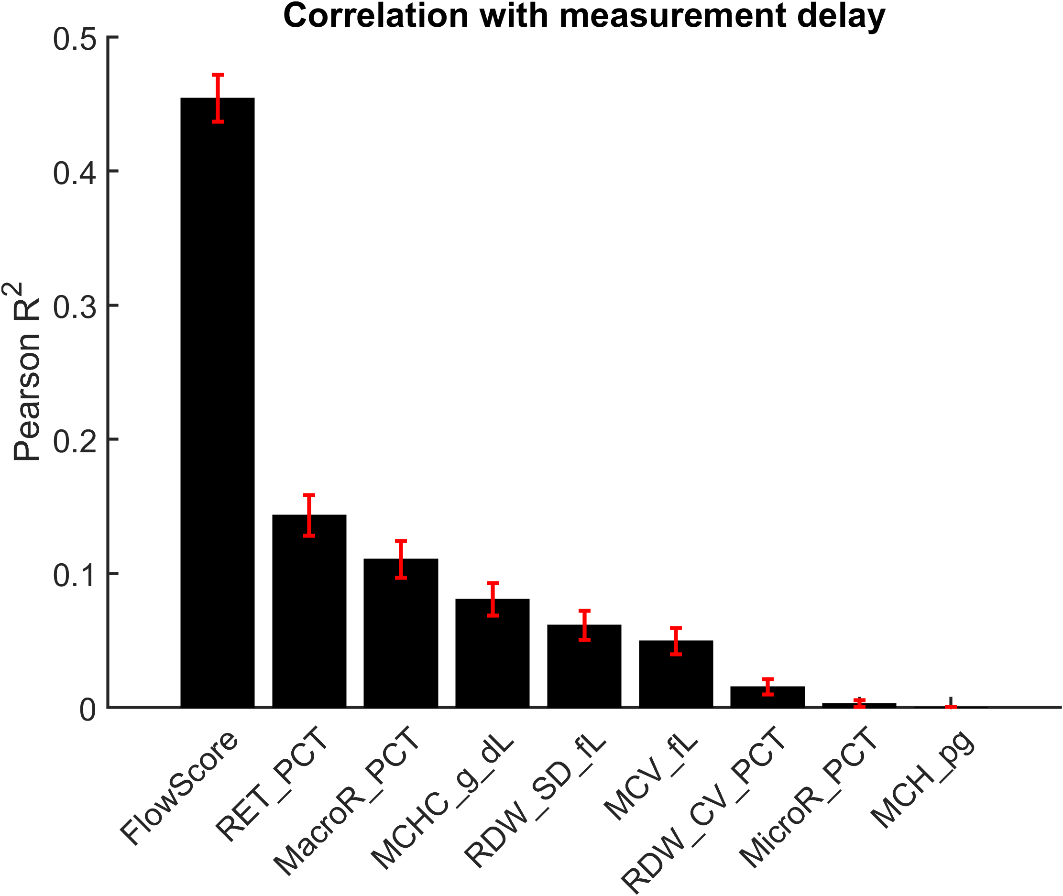
**

**Supplementary Figure S5:** Pearson’s correlation coefficient R^2^ between time delay in measurement and various RBC parameters. Error bar denotes 95% confidence interval.

**
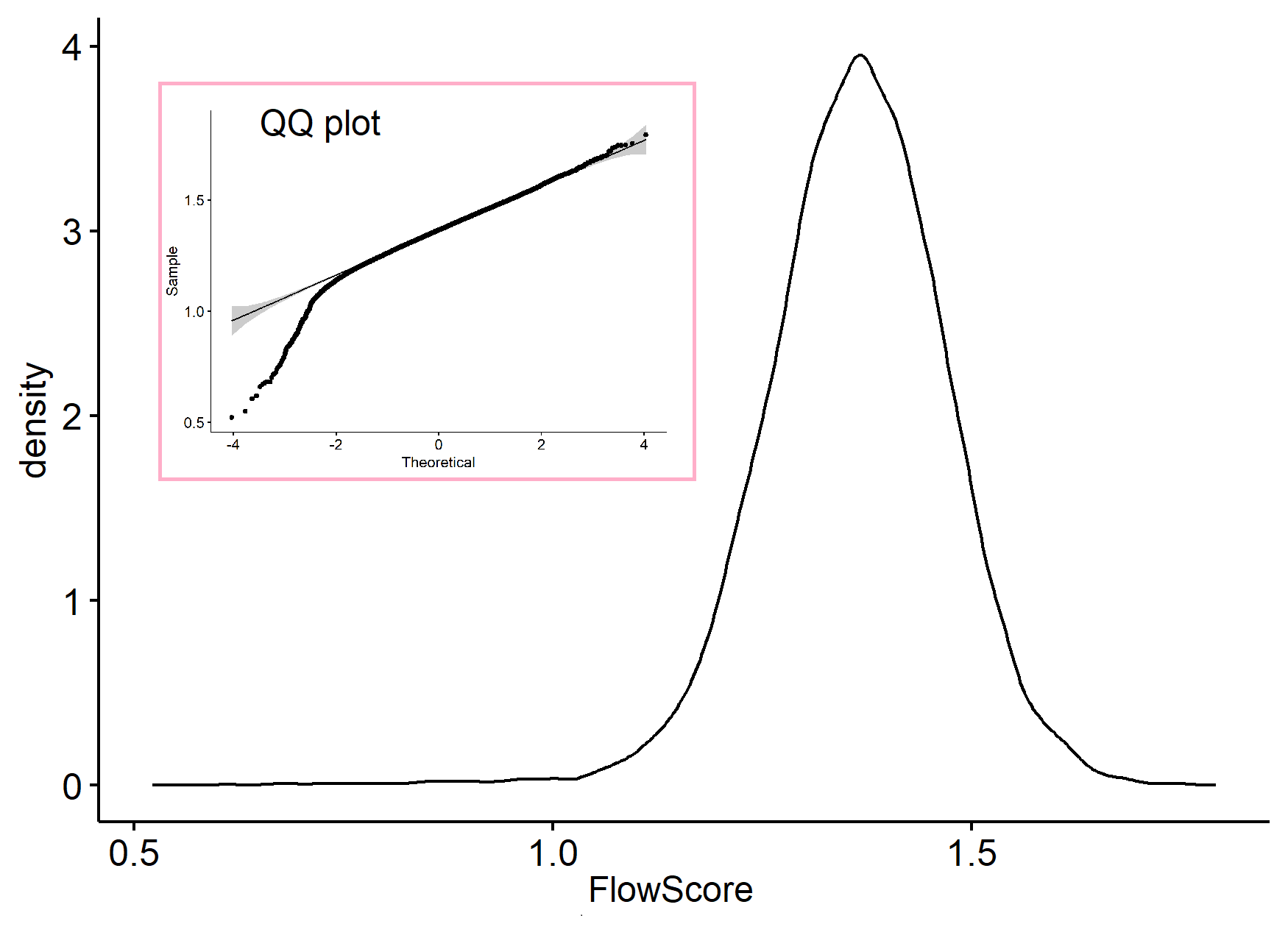
**

**Supplementary Figure S6: Normality test for FlowScore, calculated from LifeLines datasets.** Asymptotic one-sample Kolmogorov-Smirnov test: D = 0.025101, P < 0.0001. Inset shows QQ plot.
